# An approach towards azafuranomycin analogs by gold-catalyzed cycloisomerization of allenes: synthesis of (α*S*,2*R*)-(2,5-dihydro-1*H*-pyrrol-2-yl)glycine

**DOI:** 10.3762/bjoc.9.229

**Published:** 2013-09-25

**Authors:** Jörg Erdsack, Norbert Krause

**Affiliations:** 1 Organic Chemistry, Dortmund University of Technology, Otto-Hahn-Strasse 6, D-44227 Dortmund, Germany

**Keywords:** allenes, amino acids, cuprates, furanomycin, gold catalysis

## Abstract

The synthesis of (α*S*,2*R*)-(2,5-dihydro-1*H*-pyrrol-2-yl)glycine (**22**, normethylazafuranomycin) by the gold-catalyzed cycloisomerization of α-aminoallene **17** is described. The target molecule was synthesized in 13 linear steps from Cbz-protected Garner aldehyde (*R*)-**2** in an overall yield of 2.4%. The approach was first examined in model studies, which afforded the alkylated azafuranomycin derivative **13a** in 2.9% yield over 12 steps.

## Introduction

**Figure 1:**
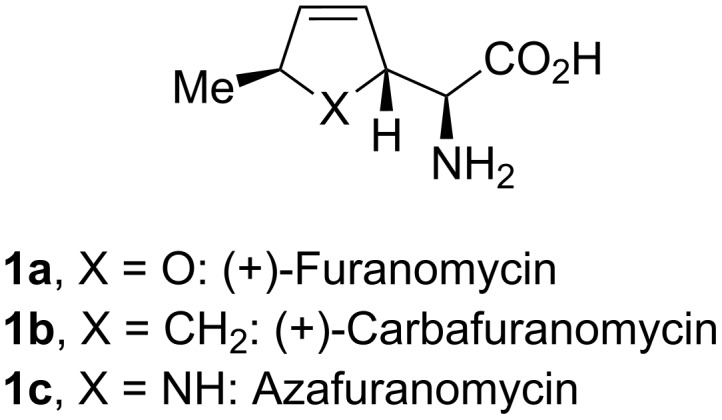
Structure of furanomycin and its carba- and aza-anolgue.

In 1967, Katagiri et al. reported the isolation of a novel antibiotic from the culture broth of the fungus *Streptomyces threomyceticus* [1]. The compound acts as a competitive antagonist for isoleucine in vitro and hampers the growth of several microorganisms, including the *E*. *coli*, *S*. *aureus* and *M*. *tuberculosis*. (+)-Furanomycin (**1a**, Figure 1, X = O) was identified as a non-proteinogenic amino acid bearing a characteristic 2,5-dihydrofuran ring. The correct (α*S*,2*R*,5*S*)-stereochemistry was established in 1980 by the first total synthesis by Joullié and co-workers [2] and the X-ray analysis of the *N*-acetyl derivative of the natural product by Shiro et al. [3]. (+)-Furanomycin belongs to the smallest natural antibiotics [4]. Therefore, the compound found considerable interest in synthetic chemistry. Until today, five total syntheses were published [2,5–8] as well as numerous reports dedicated to derivatives and stereoisomers [9–20]. Examination of structure–activity relationship (SAR) revealed a loss of antibiotic activity upon shifting of the methyl group to different positions, removal of the double bond, or change of the relative configuration [14,16]. Likewise, carbafuranomycin (**1b**) showed insufficient biological activity [17].

**Scheme 1:**
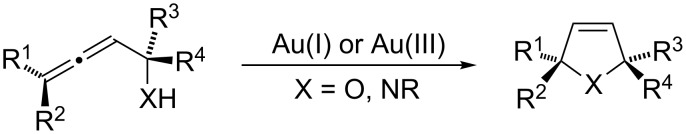
Gold-catalyzed cycloisomerization of α-functionalized allenes.

In 2007, we reported a synthesis of furanomycin derivatives by gold-catalyzed *endo*-selective cycloisomerization of α-hydroxyallenes [19]. This method opens an efficient stereoselective access to chiral 2,5-dihydrofurans by axis-to-center chirality transfer (Scheme 1) [21–32] and was applied to the total synthesis of various natural products [29–37]. Likewise, the corresponding gold-catalyzed cycloisomerization of various protected or unprotected α-aminoallenes affords 2,5-dihydropyrroles [29–32,38–39]. Due to the difference in biological activity of furanomycin (**1a**) and carbafuranomycin (**1b**), we became interested in the synthesis of derivatives of the (so far unknown) azafuranomycin (**1c**). Here, we describe the first results of this study.

## Results and Discussion

Since Boc-protected intermediates tend to decompose during late-stage oxidation to the carboxylic acid [19], we selected the Cbz-protected Garner aldehyde **2** as starting material instead of the commonly used Boc-protected analog [40–41]. We prepared aldehyde (*S*)/(*R*)-**2** on a multigram scale in three steps starting from commercial available (*S*)/(*R*)-serine methylester hydrochloride by treatment with Cbz-Cl [42], acetalization with dimethoxypropane [43], and ester reduction with DIBAL-H [44]. In our hands, this pathway was most effective as only for the reduction step Schlenk technique was necessary. Addition of lithiated *t*-butyldimethylprop-2-ynyloxysilane **3** [45] to (*S*)-**2** in THF at –78 °C in the presence of HMPA afforded alcohol **4** [46–50] in 74% yield and high *anti*-selectivity (>95:5; Scheme 2). Only traces of the *syn*-isomer were detected by TLC. Conversion of **4** into tosylate **5a** and acetate **5b** by standard conditions (*p*-TsCl/cat. DMAP in pyridine and acetic anhydride/cat. DMAP/triethylamine, respectively) proceeded in 81 and 88% yield, respectively. In contrast, treatment of alcohol **4** with diethyl chlorophosphate and *n*-BuLi or cat. DMAP in pyridine gave the phosphate **5c** in low yield. Here, direct quenching of the acetylide formed from (*S*)-**2** and lithiated **3** with diethyl chlorophosphate was more effective and afforded phosphate **5c** in 54% yield. With these propargylic electrophiles in hand, we studied the allene synthesis by copper-mediated S_N_2’-substitution [51] (Table 1, see below).

**Scheme 2:**
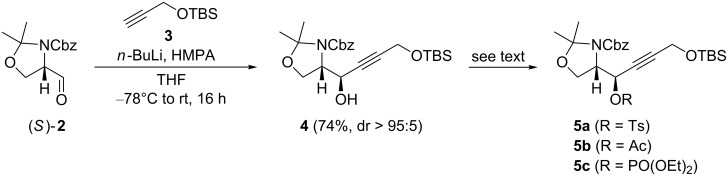
Synthesis of propargylic electrophiles **5**.

In order to establish suitable reaction conditions, we first examined the synthesis of the butyl-substituted model substrate **6a**. Treatment of propargyl tosylate **5a** with the organocopper reagent formed in situ from *n*-BuMgCl, CuBr·SMe_2_, and LiBr [52] afforded allene **6a** with up to 74% yield (Table 1, entries 1–3). In order to achieve complete conversion, a large excess of the nucleophile is required. The yield could be raised further by using the cyanocuprate *n*-BuCu(CN)MgBr [53–55] or the heterocuprate *n*-BuCu(SPh)Li [56] (Table 1, entries 4 and 5). In the latter case, no formal reduction product (**6**, R^2^ = H) was observed which might have been formed by hydrolysis of a stable copper(III) intermediate [51,56]. As expected, all S_N_2’-substitutions proceeded with excellent *anti*-stereoselectivity [51]. With propargyl acetate **5b** as starting material, allene **6a** was obtained in 85% yield using *n*-BuCu(CN)MgBr·2LiCl as nucleophile (Table 1, entry 6). In contrast, use of the heterocuprate *n*-BuCu(SPh)Li led to decomposition (Table 1, entry 7). Propargyl phosphate **5c** is also a suitable precursor of allene **6a** (Table 1, entries 8 and 9).

**Table 1:** Copper-promoted S_N_2’-substitution of propargylic electrophiles **5** to afford allenes **6**.

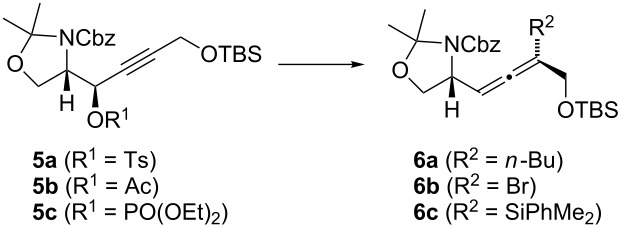				

Entry	**5**	Conditions	**6**	Yield/%

1	**5a**	4 equiv *n*-BuCuMgBr_2_·LiCl, THF, –60 → 0 °C, 90 min	**6a**	—^a^
2	**5a**	8 equiv *n*-BuCuMgBr_2_·LiCl, THF, –60 °C, 30 min	**6a**	55
3	**5a**	10 equiv *n*-BuCuMgBr_2_·LiCl, THF, –60 → 0 °C, 90 min	**6a**	74
4	**5a**	10 equiv *n*-BuCu(CN)MgBr·2LiCl, THF, –78 °C, 30 min	**6a**	83
5	**5a**	4 equiv *n*-BuCu(SPh)Li, Et_2_O, –78 °C, 30 min	**6a**	80
6	**5b**	10 equiv *n*-BuCu(CN)MgBr·2LiCl, THF, –78 °C → rt, 12 h	**6a**	85
7	**5b**	4 equiv *n*-BuCu(SPh)Li, Et_2_O, –78 °C, 30 min	**6a**	^—b^
8	**5c**	10 equiv *n*-BuCuMgBr_2_·LiCl, THF, –78 °C, 60 min	**6a**	83
9	**5c**	10 equiv *n*-BuCu(CN)MgBr·2LiCl, THF, –78 °C, 3 h	**6a**	71
10	**5a**	6 equiv LiCuBr_2_, THF, reflux, 12 h	**6b**	68
11	**5a**	1.2 equiv (PhMe_2_Si)_2_CuCNLi_2_, THF, –78 °C, 30 min	**6c**	77
12	**5b**	1.2 equiv (PhMe_2_Si)_2_CuCNLi_2_, THF, –78 °C, 30 min	**6c**	^—b^

^a^Incomplete conversion. ^b^Decomposition.

After these successful model studies, we introduced substituents into the allene which can be removed at a later stage. Treatment of propargyl tosylate **5a** with lithium dibromocuprate [57–59] or the silylcuprate (PhMe_2_Si)_2_CuCNLi_2_ [60–61] afforded the allenes **6b** and **6c** with 68 and 77% yield, respectively (Table 1, entries 10 and 11). Also these S_N_2’-substitution took place with complete *anti*-stereoselectivity. In contrast, decomposition occurred when propargyl acetate **5b** was treated with the silyl cuprate (Table 1, entry 12).

**Scheme 3:**
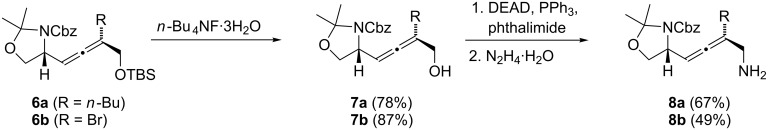
Synthesis of α-hydroxyallenes **7** and α-aminoallenes **8**.

The next steps towards the substrates of the gold-catalyzed cycloisomerization proceeded uneventfully (Scheme 3). Desilylation of allenes **6a** and **6b** with tetrabutylammonium fluoride trihydrate afforded the α-hydroxyallenes **7a**/**b** in high yield, and these were converted into the aminoallenes **8a**/**b** under standard Mitsunobu conditions (DEAD, PPh_3_, phthalimide; then hydrazine monohydrate) [38–39,62]. Unfortunately, fluoride-mediated desilylation of allene **6c** caused complete epimerization of the allenic chirality axis. Therefore, the silylallene was not used in further studies.

The results of the gold-catalyzed cycloisomerization of the allenes **7** and **8** to the five-membered heterocycles **9**/**10** are summarized in Table 2. Treatment of the α-hydroxyallene **7a** with 1 mol % AuCl_3_ in THF [21–23] afforded the desired 2,5-dihydrofuran **9a** with 84% yield (Table 2, entry 1). The temperature was decreased to 5 °C to avoid acetal cleavage by the Lewis-acidic gold catalyst [19]. For the corresponding cyclization of the bromoallene **7b**, the temperature had to be raised to 50°C in order to achieve complete conversion (Table 2, entry 2). Only traces of the acetal cleavage product were detected by TLC. However, the cycloisomerization was accompanied by partial epimerization of the allene, so that dihydrofuran **9b** was isolated as a 4:1-mixture of diastereomers.

**Table 2:** Gold-catalyzed cycloisomerization of allenes **7** and **8**.

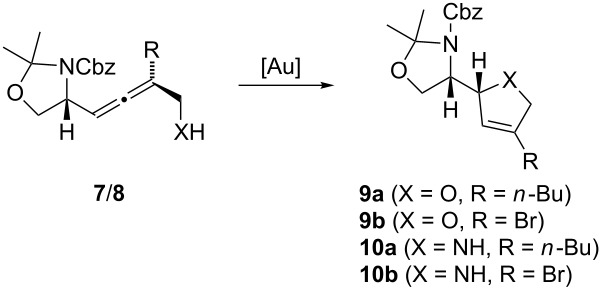				

Entry	**7**/**8**	Conditions	**9**/**10**	Yield/%

1	**7a**	1 mol % AuCl_3_,THF, 5 °C, 12 h	**9a**	84
2	**7b**	2 mol % AuCl_3_, THF, 50 °C, 5 h	**9b**	77^a^
3	**8a**	10 mol % AuCl, 10 mol % imidazole, DCE, 80 °C, 12 h	**10a**	66^b^
4	**8a**	10 mol % AuCl, 10 mol % imidazole, toluene, 80 °C, 12 h	**10a**	78
5	**8b**	7 mol % Ph_3_PAuCl, 7 mol % AgBF_4_, 7 mol % imidazole, toluene, 100 °C, 12 h	**10b**	47^c^

^a^dr = 4:1. ^b^The *N*-chloroethylated dihydropyrrole was formed as side product (29% yield). ^c^Yield of the twofold protected dihydropyrrole **11b** obtained by treatment of **10b** with CbzCl and DMAP; 2’-*epi*-**11b** was obtained as minor product (4% yield).

As expected, the cycloisomerization of allenes **8** bearing an unprotected amino group is much slower [38–39] and requires rather forcing conditions. For a complete conversion of α-aminoallene **8a**, 10 mol % of AuCl [38–39], 10 mol % of imidazole as stabilizing ligand and an elevated temperature (80 °C) are necessary. With dichloroethane as solvent, dihydropyrrole **10a** was obtained in 66% yield (Table 2, entry 3); however, this was accompanied by 29% of the corresponding *N*-chloroethylated product. This undesired side product could be avoided by using toluene as the solvent (Table 2, entry 4). Application of these conditions to the brominated α-aminoallene **8b** gave incomplete conversion. Full conversion was achieved with 7 mol % each of Ph_3_PAuCl, AgBF_4_ and imidazole in toluene at 100 °C (Table 2, entry 5). The dihydropyrrole **10b** thus formed could not purified completely even after repeated column chromatography. Therefore, the crude product was treated with CbzCl and DMAP [63] to give the twofold protected dihydropyrrole **11b** (formula not shown) with 47% yield over two steps. Additionally, we isolated the epimeric compound 2’-*epi*-**11b** in 4% yield, indicating a minimal epimerization of bromoallene **8b** during the gold-catalyzed cyclization.

**Scheme 4:**
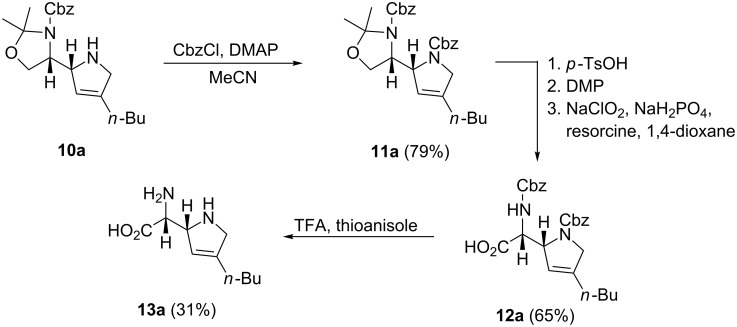
Synthesis of azafuranomycin analog **13a**.

**Scheme 5:**
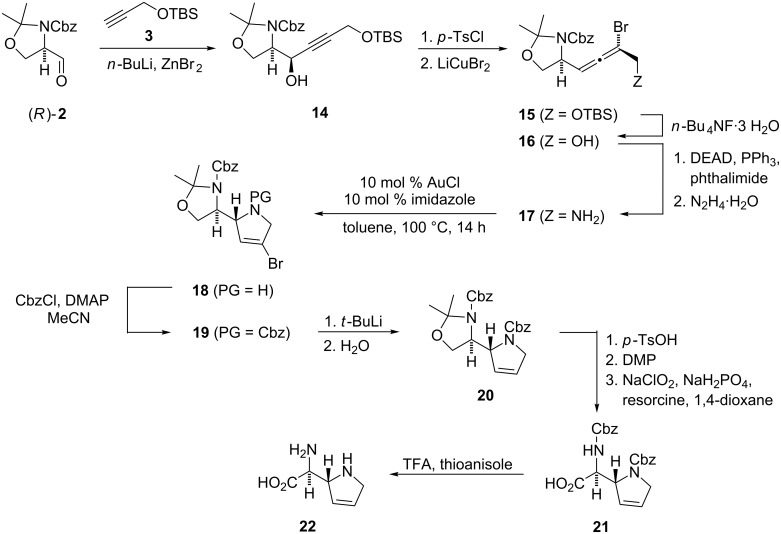
Synthesis of (α*S*,2*R*)-(2,5-dihydro-1*H*-pyrrol-2-yl)glycine (**22**).

The synthesis of azafuranomycin analogs was continued with the twofold Cbz-protected heterocycle **11a** which was obtained with 79% yield by treatment of **10a** with CbzCl and DMAP [63] (Scheme 4). This protection step was carried out in order to avoid dehydrogenation or chlorination of the secondary amine in the subsequent oxidation steps [64–65]. Acetal cleavage under mild protic conditions furnished the hydroxycarbamate, which underwent two-step oxidation with Dess–Martin periodinane [66–67] and sodium chlorite in buffered solution [68] in the presence of resorcine [19]. The carboxylic acid **12a** was isolated in 65% yield. Finally, the protecting groups were removed with trifluoracetic acid in the presence of thioanisole [69] to afford the azafuranomycin analog **13a** with 31% yield. Later, we found that a higher excess of thioanisole, which captures benzylic cations in the deprotection step, affords higher yields of the amino acid (Scheme 5).

After successful conclusion of the model studies, the synthesis of (α*S*,2*R*)-(2,5-dihydro-1*H*-pyrrol-2-yl)glycine (**22**, normethylazafuranomycin) was carried out (Scheme 5). The aldehyde (*R*)-**2**, which was prepared from D-serine [42–44], underwent chelation-controlled nucleophilic addition of metallated alkyne **3** [45] to give the propargyl alcohol **14** in 84% yield and a *syn*-diastereoselectivity of >95:5 [7,46–47]. After tosylation of **14** (85% yield), synthesis of the bromoallene **15** with lithium dibromocuprate resulted in an unexpected epimerization of the allene moiety to afford an inseparable 2:1-mixture of diastereomers in 66% yield. Addition of stabilizing ligands ((*n*-Bu)_3_P or (*n*-BuO)_3_P) did not affect this loss of stereoselectivity. Fortunately, the correct stereoisomer was enriched at the stage of the dihydropyrrole **18** due to several purification steps.

Desilylation of **15** with tetrabutylammonium fluoride trihydrate (94% yield) and conversion of **16** into the α-aminoallene **17** under Mitsunobu conditions (45% yield) [38–39,62] set the stage for the gold-catalyzed cycloisomerization. This was carried out with 10 mol % each of AuCl and imidazole in toluene at 100 °C to give the desired dihydropyrrole **18** in ca. 67% yield. Similar to the diastereomer **10b**, compound **18** could not be purified in a sufficient manner even after repeated column chromatography. Treatment of **18** with CbzCl/DMAP [63] gave the fully protected heterocycle **19** in 42% yield over two steps from **17**. The spectroscopic data are identical with those of 2’-*epi*-**11b**, except for the sign of the optical rotation {**19**: [α]^19^_D_ +37.9 (*c* 1.27, CHCl_3_); 2’-*epi*-**11b**: [α]^20^_D_ –47.0 (*c* 0.10, CHCl_3_)}.

For the debromination of dihydropyrrole **19**, we first tested radical conditions ((*n*-Bu)_3_SnH/AIBN), but these led to complete decomposition. This is surprising since carbamates are known to be stable under radical conditions [70]. Indeed, treatment of diastereomer **11b** with (*n*-Bu)_3_SnH/AIBN afforded the desired dehalogenated dihydropyrrole with 66% yield (formula not shown). For the conversion of **19** to **20**, we applied a bromine–lithium exchange with 2 equivalents of *t*-BuLi in diethyl ether at –90 °C [71], followed by hydrolysis. Even though oxazolidines are known to be sensitive towards organolithium compounds [19,72–74], dehalogenated dihydropyrrole **20** was obtained in 60% yield, together with 22% of reisolated **19**. The remaining steps towards azafuranomycin analog **22** followed those established for **13a**: acetal cleavage (71% yield), two-step oxidation which afforded protected amino acid **21** with 72% yield [19,66–68], and final deprotection according to the procedure of Kiso et al. (50 equiv thioanisole and 270 equiv TFA per Cbz-group) [69] gave the target molecule **22** with 66% yield after purification by ion exchange chromatography (DOWEX 50W X8).

## Conclusion

We have developed the first synthesis of the azafuranomycin analog (α*S*, 2*R*)-(2,5-dihydro-1*H*-pyrrol-2-yl)glycine (**22**) in 13 linear steps with an overall yield of 2.4% starting from the Cbz-protected Garner aldehyde (*R*)-**2**. The key step is the gold-catalyzed cycloisomerization of α-aminoallene **17** to dihydropyrrole **18**. The sequence was first tested in model studies which afforded butyl-substituted azafuranomycin derivative **13a** in 12 linear step with an overall yield of 2.9% starting from (*S*)-**2**.

## Supporting Information

File 1 Experimental part.

## References

[1] Katagiri K, Tori K, Kimura Y, Yoshida T, Nagasaki T, Minato H (1967). J Med Chem.

[2] Semple J E, Wang P C, Lysenko Z, Joullié M M (1980). J Am Chem Soc.

[3] Shiro M, Nakai H, Tori K, Nishikawa J, Yashimura Y, Katagiri K (1980). J Chem Soc, Chem Commun.

[4] von Nussbaum F, Brands M, Hinzen B, Weigand S, Häbich D (2006). Angew Chem, Int Ed.

[5] Kang S H, Lee S B (1998). Chem Commun.

[6] Zhang J H, Clive D L J (1999). J Org Chem.

[7] VanBrunt M P, Standaert R F (2000). Org Lett.

[8] Zimmermann P J, Blanarikova J, Jäger V (2000). Angew Chem, Int Ed.

[9] Masamune T, Ono M (1975). Chem Lett.

[10] Divanfard H R, Lysenko Z, Semple J E, Wang P-C, Joullié M M (1981). Heterocycles.

[11] Robins M J, Parker J M R (1983). Can J Chem.

[12] Wiliams R M, Sinclair P J, Zhai D, Chen D (1988). J Am Chem Soc.

[13] Braithwaite D H, Holzapfel C W, Wiliams D B G (1999). J Chem Res, Synop.

[14] Kazmaier U, Pähler S, Endermann R, Häbich D, Kroll H-P, Riedl B (2002). Bioorg Med Chem.

[15] Chattopadhyay S K, Sarkar K, Karmakar S (2005). Synlett.

[16] Zimmermann P J, Lee J Y, Hlobilova (neé Blanarikova) I, Endermann R, Häbich D, Jäger V (2005). Eur J Org Chem.

[17] Lee J Y, Schiffer G, Jäger V (2005). Org Lett.

[18] Jirgensons A, Marinozzi M, Pellicciari R (2005). Tetrahedron.

[19] Erdsack J, Krause N (2007). Synthesis.

[20] Avenoza A, Busto J H, Canal N, Corzana F, Peregrina J M, Pérez-Fernández M, Rodríguez F (2010). J Org Chem.

[21] Hoffmann-Röder A, Krause N (2001). Org Lett.

[22] Krause N, Hoffmann-Röder A, Canisius J (2002). Synthesis.

[23] Deutsch C, Gockel B, Hoffmann-Röder A, Krause N (2007). Synlett.

[24] Aksιn Ö, Krause N (2008). Adv Synth Catal.

[25] Winter C, Krause N (2009). Green Chem.

[26] Asikainen M, Krause N (2009). Adv Synth Catal.

[27] Aksın-Artok Ö, Krause N (2011). Adv Synth Catal.

[28] Minkler S R K, Lipshutz B H, Krause N (2011). Angew Chem, Int Ed.

[29] Krause N, Belting V, Deutsch C, Erdsack J, Fan H-T, Gockel B, Hoffmann-Röder A, Morita N, Volz F (2008). Pure Appl Chem.

[30] Krause N, Aksin-Artok Ö, Breker V, Deutsch C, Gockel B, Poonoth M, Sawama Y, Sawama Y, Sun T, Winter C (2010). Pure Appl Chem.

[31] Krause N, Winter C (2011). Chem Rev.

[32] Krause N, Aksin-Artok Ö, Asikainen M, Breker V, Deutsch C, Erdsack J, Fan H-T, Gockel B, Minkler S, Poonoth M (2012). J Organomet Chem.

[33] Volz F, Krause N (2007). Org Biomol Chem.

[34] Volz F, Wadman S H, Hoffmann-Röder A, Krause N (2009). Tetrahedron.

[35] Miura T, Shimada M, de Mendoza P, Deutsch C, Krause N, Murakami M (2009). J Org Chem.

[36] Sun T, Deutsch C, Krause N (2012). Org Biomol Chem.

[37] Gao Z, Li Y, Cooksey J P, Snaddon T N, Schunk S, Viseux E M E, McAteer S M, Kocienski P J (2009). Angew Chem, Int Ed.

[38] Morita N, Krause N (2004). Org Lett.

[39] Morita N, Krause N (2006). Eur J Org Chem.

[40] Garner P (1984). Tetrahedron Lett.

[41] Garner P, Park J M (1987). J Org Chem.

[42] Hassall C H, Thomas J O (1968). J Chem Soc C.

[43] Chhabra S R, Mahajan A, Chan W C (2002). J Org Chem.

[44] McKillop A, Taylor R J K, Watson R J, Lewis N (1994). Synthesis.

[45] Tsou H-R, Mamuya N, Johnson B D, Reich M F, Gruber B C, Ye F, Nilakantan R, Shen R, Discafani C, DeBlanc R (2001). J Med Chem.

[46] Herold P (1988). Helv Chim Acta.

[47] Gruza H, Kiciak K, Krasiński A, Jurczak J (1997). Tetrahedron: Asymmetry.

[48] Chun J, Byun H-S, Bitman R (2003). J Org Chem.

[49] Masuda Y, Mori K (2005). Eur J Org Chem.

[50] Erdsack J, Schürmann M, Preut H, Krause N (2008). Acta Crystallogr, Sect E: Struct Rep Online.

[51] Krause N, Hoffmann-Röder A (2004). Tetrahedron.

[52] Elsevier C J, Vermeer P (1989). J Org Chem.

[53] Yanagisawa A, Noritake Y, Nomura N, Yamamoto H (1991). Synlett.

[54] Yanagisawa A, Nomura N, Yamamoto H (1993). Synlett.

[55] Torneiro M, Fall Y, Castedo L, Mouriño A (1997). J Org Chem.

[56] Nantz M H, Bender D M, Janaki S (1993). Synthesis.

[57] Montury M, Goré J (1980). Synth Commun.

[58] Elsevier C J, Vermeer P, Gedanken A, Runge W (1985). J Org Chem.

[59] Boukouvalas J, Pouliot M, Robichaud J, MacNeil S, Snieckus V (2006). Org Lett.

[60] Jin J, Weinreb S M (1997). J Am Chem Soc.

[61] Aoyagi S, Hirashima S, Saito K, Kibayashi C (2002). J Org Chem.

[62] Mitsunobu O (1981). Synthesis.

[63] Ashley E R, Cruz E, Stoltz B M (2003). J Am Chem Soc.

[64] Nicolaou K C, Mathison C J N, Montagnon T (2004). J Am Chem Soc.

[65] Hayes C J, Sherlock A E, Selby M D (2006). Org Biomol Chem.

[66] Dess D B, Martin J C (1983). J Org Chem.

[67] Boeckmann R K, Shao P, Mullins J (2004). Org Synth.

[68] Bal B S, Childers W E, Pinnick H W (1981). Tetrahedron.

[69] Kiso Y, Ukawa K, Akita T (1980). J Chem Soc, Chem Commun.

[70] Muratake H, Natsume M, Nakai H (2006). Tetrahedron.

[71] Neumann H, Seebach D (1978). Chem Ber.

[72] Reginato G, Mordini A, Degl’Innocenti A, Caracciolo M (1995). Tetrahedron Lett.

[73] Reginato G, Mordini A, Caracciolo M (1997). J Org Chem.

[74] Govek S P, Overman L E (2007). Tetrahedron.

