# Supplementary material for: An approach towards azafuranomycin analogs by gold-catalyzed cycloisomerization of allenes: synthesis of (αS,2R)-(2,5-dihydro-1H-pyrrol-2-yl)glycine
Source: Beilstein J Org Chem. 2013 Sep 25;9:1936–42. doi: 10.3762/bjoc.9.229 (PMC3817573; doi:10.3762/bjoc.9.229)
Supplement: File 1 — Experimental part. [file Beilstein_J_Org_Chem-09-1936-s001.pdf]

Supporting Information

for

An approach towards azafuranomycin analogs by gold-catalyzed cycloisomerization of allenes: synthesis of (α*S*,2*R*)-(2,5-dihydro-1*H*-pyrrol-2-yl)glycine

Jörg Erdsack and Norbert Krause*

Address: Organic Chemistry, Dortmund University of Technology, Otto-Hahn-Strasse 6, D-44227 Dortmund, Germany

Email: Norbert Krause – norbert.krause@tu-dortmund.de
* Corresponding author

Experimental part

General information: Melting points were determined with a Reichert thermovar mp apparatus and are uncorrected. IR spectra were obtained with a Nicolet Avatar 320 FTIR spectrometer either in KBr pellets or as liquid film between NaCl plates. ^{1}H and ^{13}C NMR spectra were recorded with a Bruker DRX 400 or a Bruker DRX 500 spectrometer using the signals of the undeuterated solvent as the standard. Chemical shifts were determined relative to the residual solvent peaks ($CHCl_3$: δ = 7.26 for protons, δ = 77.0 for carbon atoms; CD_3OD: δ = 3.31 for protons, δ = 49.05 for carbon atoms; D_2O: δ = 4.81 for protons; C_6D_6: δ = 7.15 for protons, δ = 128.02 for carbon atoms). Carbon atoms were assigned with APT experiments. The products bearing a Cbz group often show broadened and/or duplicated peaks in NMR spectra

due to slow conformational inversion. In diastereoisomeric mixtures, the peaks of the main isomer were assigned with an asterisk (*), if possible. ESI spectra were measured with a LTQ ORBITRAP equipped with a Hypersil gold column (diameter 50 × 1 mm, particle size 1.9 µm). Optical rotations were determined with a Perkin-Elmer 341 polarimeter. All reactions were carried out under an argon atmosphere in oven-dried glassware. THF and diethyl ether was distilled from sodium/benzophenone, CH_2Cl_2, DMF, HMPA and benzene were distilled from CaH_2 prior to use. TLC plates were visualized by immersion in a solution of alkaline permanganate (2 g $KMnO_4$ and 10 g Na_2CO_3 in 200 mL H_2O), followed by heating. Silica gel (particle size 0.035–0.070 mm) was purchased from ACROS. DOWEX 50W X8 (H^+, 200–400 mesh) and Amberlite IR-120 cation exchange resin (H^+ form, 16–45 mesh) were purchased by Fluka. *n*-BuMgCl was titrated with salicylaldehyde phenylhydrazone according to the procedure of Love and Jones [1]; *n*-BuLi and *t*-BuLi were titrated with diphenylacetic acid according to the procedure by Kofron and Baclawski [2]. AuCl was purchased from Acros and was used as received. An $AuCl_3$ stock solution was prepared by dissolving the gold salt in the appropriate volume of anhydrous acetonitrile in a Schlenk tube under argon and cooling with ice. Preparation of the $ZnBr_2$ stock solution: Anhydrous $ZnBr_2$ was transferred in a Schlenk tube equipped with a magnetic stirring bar. The tube was evacuated and heated to 300–350 °C for several minutes with a heating gun. After cooling, the tube was flushed slowly with argon and anhydrous diethyl ether was added (100 mL per 100 mmol $ZnBr_2$). The suspension was stirred overnight to effect dissolution. The supernatant solution can be used directly for synthesis (note: the salt was not completely dissolved). Purification of amino acids by ion exchange chromatography and activation of cation exchange resins: The resin was transferred into a glass column (diameter 1.5 cm, height: 10 cm per 25 mg expected product) and was purged successively with distilled water, 1 N

NH_4OH, distilled water until pH 7, 1 N HCl, distilled water until pH 7, methanol and distilled water. The crude product mixture from the deprotecting step was taken up in distilled water or water/methanol mixture (depending on the solubility) and put on the column. The column was purged successively with distilled water, methanol and distilled water to remove impurities. Then, the amino acid was eluted with 0.5 N NH_4OH. Fractions were spotted on TLC plates, and those giving a positive ninhydrine test were pooled and concentrated carefully under reduced pressure (rotatory evaporator with ≈60 °C bath temperature).

(4*S*,1'*R*)-3-Benzyloxycarbonyl-4-[4-(*tert*-butyldimethylsilyloxy)-1-hydroxybut-2'-inyl]-2,2-dimethyloxazolidine (4). In an oven-dried three-necked 500 mL flask equipped with a magnetic stirring bar and nitrogen inlet, **3** [3] (6.80 g, 40.0 mmol) was dissolved in anhydrous THF (150 mL) under argon. After cooling to –30 °C, *n*-BuLi (16.0 mL, 40.0 mmol, 2.5 M in hexane) was added slowly dropwise *via* syringe and stirring was continued for 15 min at –30 °C. HMPA (7.0 mL, 40.0 mmol) was added *via* syringe and the solution was cooled to –78 °C. A solution of aldehyde (*S*)-**2** [4–6] (5.30 g, 20.1 mmol) in anhydrous THF (15 mL) was added slowly by syringe on the inner surface of the flask. The mixture was kept at –78 °C for 2 h and was allowed to warm to rt overnight (~12 h), after which TLC control showed complete conversion of the starting material. The mixture was poured in aq sat. NH_4Cl (250 mL) and the organic phase was separated. The residue was diluted with water and was extracted with diethyl ether (3 × 80 mL). The combined organic layers were washed with brine, dried ($MgSO_4$), filtered, and the solvent was evaporated. The residue was purified by column chromatography (silica gel, isohexane/EtOAc 85:15 → 4:1) to give the title compound **4** (6.41 g, 74% yield) as a yellow oil (*anti*:*syn* > 95:5, by NMR analysis). $[\alpha]^{24}_{D}$ –18.2 (*c* 1.20, $CHCl_3$); R_f = 0.66 (isohexane/EtOAc 7:3); ^1H NMR (400 MHz,

$CDCl_3$): δ = 7.33 (m, 5H), 5.15–5.09 (br m, 2H), 4.68 (br s, 1H), 4.31–4.01 (m, 5H), 1.64 / 1.57 / 1.51 / 1.47 (4 × s, 6H), 0.87 (s, 9H), 0.08 (s, 6H); ^{13}C NMR (100 MHz, $CDCl_3$): δ = 154.2, 152.0 (2 NCO_2Bn), 136.0, 135.6 (2 Cq, arom.), 128.4, 128.2, 128.1, 127.9, 127.7 (5 CH, arom.), 95.1 [$C(CH_3)_2$], 84.8, 84.5 (2 C≡C), 83.0, 82.4 (2 C≡C), 67.5, 66.7 (2 CH_2), 64.8, 64.7, 64.5 (3 CH_2), 63.0, 62.5, 62.4, 60.7 (4 CH), 51.4 (CH_2), 25.7 [$C(CH_3)_2$], 25.6 [$SiC(CH_3)_3$], 25.4, 25.1, 23.0 [3 $C(CH_3)_2$], 18.1 [$SiC(CH_3)_3$], –5.3 [$Si(CH_3)_2$]; IR (film): 3437 (br), 3066, 3034, 2954, 2930, 2885, 2858, 1705, 1498, 1471, 1410, 1383, 1353, 1312, 1258, 1211, 1155, 1131, 1091, 837, 815, 779, 698 cm^{-1}; HRMS (ESI): *m/z* [*M* + H]$^+$ calcd for $C_{23}H_{36}NO_5{}^{28}Si$: 434.2357; found: 434.2360.

(4*S*,1'*R*)-3-Benzyloxycarbonyl-4-[4-(*tert*-butyldimethylsilyloxy)-1-(toluene-4-sulfonyloxy)but-2-inyl]-2,2-dimethyloxazolidine (5a). In an oven-dried Schlenk tube equipped with a magnetic stirring bar, alcohol **4** (1.45 g, 3.34 mmol) was dissolved in a mixture of anhydrous CH_2Cl_2 (16 mL) and anhydrous pyridine (14 mL) under argon. DMAP (41 mg, 0.33 mmol) and *p*-TsCl (3.83 g, 20.06 mmol) were added in one portion and the solution was allowed to stir at rt for 16 h, after which TLC control showed complete conversion of the starting material. The mixture was poured in aq sat. $NaHCO_3$ (100 mL) and the mixture was stirred vigorously until the gas evolution was finished (~5 min). The organic phase was separated and the aqueous layer was extracted with diethyl ether (4 × 50 mL). The combined organic layers were washed with brine, dried ($MgSO_4$), filtered and the solvent was evaporated. The residue was purified by column chromatography (silica gel, isohexane/EtOAc 85:15) to give the title compound **5a** (1.59 g, 81% yield) as a colorless oil. $[\alpha]^{24}_D$ –46.8 (*c* 1.05, $CHCl_3$); R_f = 0.44 (isohexane/EtOAc 4:1); ^{1}H NMR (400 MHz, $CDCl_3$): δ = 7.80 (d, 1H), 7.71 (d, 1H), 7.38–7.26 (m, 7H), 5.57 (d, 1H),

5.18–5.04 (m, 2H), 4.23–4.00 (m, 5H), 2.41 (d, 3H), 1.59 / 1.56 / 1.50 / 1.42 (4 × s, 6H), 0.85 (d, 9H), 0.02 (d, 6H); ^{13}C NMR (100 MHz, $CDCl_3$): δ = 153.0, 151.7 (2 NCO_2Bn), 144.7, 144.6 (2 Cq, arom.), 136.0, 135.9 (2 Cq, arom.), 133.7, 133.6 (2 Cq, arom.), 129.6, 129.5, 128.6, 128.3, 128.1, 127.9 (6 CH, arom.), 95.8, 95.2 [2 $C(CH_3)_2$], 88.5, 88.3 (2 C≡C), 77.7 (C≡C), 69.5, 68.8 (2 CH), 67.4, 67.0, 64.1, 63.4 (4 CH_2), 60.8, 59.6 (2 CH), 51.2 (CH_2), 26.0 [$C(CH_3)_2$], 25.6 [$SiC(CH_3)_3$], 25.3, 24.9, 23.3 [3 $C(CH_3)_2$], 21.6 (SO_2-C_6H_4-CH_3), 18.1 [$SiC(CH_3)_3$], –5.3 [$Si(CH_3)_2$]; IR (film): 3066, 3033, 2954, 2930, 2885, 2858, 1715, 1598, 1498, 1463, 1406, 1383, 1352, 1305, 1259, 1211, 1190, 1178, 1142, 1096, 1040, 1006, 931, 837, 814, 780, 748, 699, 665, 563 cm^{-1}; HRMS (ESI): *m/z* $[M + H]^+$ calcd for $C_{30}H_{41}NO_7{}^{32}S^{28}Si$: 588.2446; found 588.2443.

(4*S*,1'*R*)-3-Benzyloxycarbonyl-4-[1-acetoxy-4-(*tert*-butyldimethylsilyloxy)-but-2-inyl]-2,2-dimethyloxazolidine (5b). In an oven-dried Schlenk tube equipped with a magnetic stirring bar, alcohol **4** (340 mg, 0.78 mmol), triethylamine (220 µL, 1.57 mmol) and DMAP (5 mg, 0.04 mmol) were dissolved in anhydrous CH_2Cl_2 (7 mL) under argon. After cooling to –35 °C, acetic anhydride (120 µL, 1.18 mmol) was added dropwise and the solution was allowed to stir for 90 min with warming to –20 °C. Aq. sat. $NaHCO_3$ (10 mL) was added and the mixture was stirred for 20 min at rt. The organic phase was separated and the aqueous layer was extracted with CH_2Cl_2 (2 × 10 mL). The combined organic layers were washed with brine, dried ($MgSO_4$), filtered and the solvent was evaporated. The residue was purified by column chromatography (silica gel, isohexane/EtOAc 85:15) to give the title compound **5b** (329 mg, 88% yield) as a colorless oil. $[\alpha]^{24}_D$ –50.6 (*c* 1.00, $CHCl_3$); R_f = 0.60 (isohexane/EtOAc 4:1); ^1H NMR (400 MHz, $CDCl_3$): δ = 7.34–7.28 (m, 5H), 5.88 (d, 1H), 5.17–5.03 (m, 2H), 4.28–4.02 (m, 5H), 2.04 (d, 3H), 1.56 / 1.48 / 1.41 (3

× s, 6H), 0.86 (d, 9H), 0.07 (d, 6H); ^{13}C NMR (100 MHz, $CDCl_3$): δ = 169.4, 169.3 (2 H_3CCO_2), 152.8, 151.9 (2 NCO_2Bn), 135.9, 135.8 (2 Cq, arom.), 128.4, 128.0, 127.9 (3 CH, arom.), 95.2, 95.0 [2 $C(CH_3)_2$], 85.7, 85.4 (2 C≡C), 79.6, 79.5 (2 C≡C), 67.2, 66.9 (2 CH_2), 64.4, 63.8 (2 CH_2), 62.8, 62.3 (2 CH), 60.2, 59.1 (2 CH), 51.3 (CH_2), 26.3 [$C(CH_3)_2$], 25.6 [$SiC(CH_3)_3$], 24.5, 23.0, 20.8 [3 $C(CH_3)_2$], 18.0 [$SiC(CH_3)_3$], –5.3 [$Si(CH_3)_2$]; IR (film): 3066, 3034, 2955, 2932, 2885, 2858, 1752, 1713, 1498, 1405, 1383, 1351, 1307, 1253, 1226, 1143, 1017, 962, 837, 779 cm^{-1}; HRMS (ESI): *m/z* [*M* + H]$^+$ calcd for $C_{25}H_{38}NO_6{}^{28}Si$: 476.2463; found 476.2462.

(4*S*,1’*R*)-3-Benzyloxycarbonyl-4-[4-(*tert*-butyldimethylsilyloxy)-1-(diethoxy-phosphoryloxy)but-2-inyl]-2,2-dimethyloxazolidine (5c). In an oven-dried Schlenk tube equipped with a magnetic stirring bar, **3** [3] (1.08 g, 6.34 mmol) was dissolved in anhydrous THF (22 mL) under argon. After cooling to –25 °C, *n*-BuLi (2.54 mL, 6.34 mmol, 2.5 M in hexane) was added slowly dropwise via syringe and stirring was continued for 15 min at –30 °C. After cooling to –78 °C, HMPA (1.14 g, 6.34 mmol) was added via syringe. After 5 min, a solution of aldehyde (*S*)-**2** [4–6] (840 mg, 3.17 mmol) in anhydrous THF (3 mL) was added slowly by syringe on the inner surface of the flask. The mixture was kept at –78 °C for 60 min and was allowed to warm to rt overnight (~12 h), after which TLC control showed complete conversion of the starting material. After cooling to 0 °C, diethylchlorophosphate (1.94 mL, 2.30 g, 12.7 mmol) was added via syringe and the cooling bath was removed. After stirring for 60 min at rt, the mixture was poured in aq sat. NH_4Cl (50 mL) and the organic phase was separated. The residue was extracted with diethyl ether (3 × 30 mL). The combined organic layers were washed with brine, dried ($MgSO_4$), filtered, and the solvent was evaporated. The residue was purified by column chromatography (silica gel, isohexane/EtOAc 7:3) to give the title compound **5c** (970 mg, 54% yield) as a

yellow oil (*anti*:*syn* > 95:5, by NMR analysis). $[\alpha]^{20}_{D}$ –40.3 (*c* 0.98, $CHCl_3$); R_f = 0.39 (isohexane/EtOAc 3:2); ^1H NMR (400 MHz, $CDCl_3$): δ = 7.34 – 7.28 (m, 5H), 5.88 (d, 1H), 5.17–5.03 (m, 2H), 4.28–4.02 (m, 5H), 2.04 (d, 3H), 1.56 / 1.48 / 1.41 (3 × s, 6H), 0.86 (br s, 9H), 0.07 (d, 6H); ^{13}C NMR (100 MHz, $CDCl_3$): δ = 152.9, 151.9 (2 NCO_2Bn), 135.9, 135.8 (2 Cq, arom.), 128.4, 127.9 (2 CH, arom.), 95.4, 94.8 [2 $C(CH_3)_2$], 86.9, 86.7 (2 C≡C), 79.3 (C≡C), 67.1, 66.8 (2 CH_2), 66.5, 65.5 (2 CH), 64.1, 63.9, 63.8, 63.8, 63.4 (5 CH_2), 61.1, 60.0 (2 CH), 51.3 (CH_2), 26.0 [$C(CH_3)_2$], 25.5 [$SiC(CH_3)_3$], 25.2, 24.8, 23.3 [3 $C(CH_3)_2$], 18.0 [$SiC(CH_3)_3$], 15.9, 15.8 ($POCH_2CH_3$), –5.4 [$Si(CH_3)_2$]; IR (film): 3065, 3034, 2984, 2932, 2858, 1712, 1463, 1407, 1384, 1352, 1306, 1264 (s, P=O), 1211, 1141, 1088, 1039, 1001, 887, 779 cm^{-1}; HRMS (ESI): *m/z* $[M + H]^+$ calcd for $C_{27}H_{45}NO_{8+}P^{28}Si$: 570.2647; found: 570.2657.

(4*R*,2'*S*)-3-Benzyloxycarbonyl-4-(3'-(*tert*-butyldimethylsilyloxymethyl)hepta-1',2'-dienyl)-2,2-dimethyloxazolidine (6a). Anhydrous LiCl (1.01 g, 23.8 mmol) was transferred into a 250 mL flask equipped with a magnetic stirring bar and nitrogen inlet. The flask was evacuated and heated with a heating gun to ~300 °C for several minutes. After cooling, the flask was flushed slowly with argon and CuCN (1.07 g, 11.9 mmol) and anhydrous THF (45 mL) were added. The suspension was stirred at rt until the salts were dissolved, cooled to –78 °C, and *n*-BuMgCl (6.96 mL, 11.9 mmol, 20% in THF/toluene) was added dropwise. The mixture was warmed to rt for 10 min and then cooled again to –78 °C. A solution of tosylate **5a** (0.70 g, 1.19 mmol) in anhydrous THF (7 mL) was added slowly by syringe on the inner surface of the flask. After stirring for 10 min, TLC control indicated complete conversion of the starting material. The reaction was quenched by addition of aq sat. NH_4Cl (7 mL) and the cooling bath was removed. After stirring at rt for 90 min, the supernatant solution was poured into isohexane (250 mL) to afford a suspension. The residue was treated

with isohexane/diethyl ether (1:1, 100 mL), and both suspensions were stirred for 30 min. After filtration through a short silica pad, the solvent was removed, and the residue was purified by column chromatography (silica gel, isohexane/EtOAc 93:7) to afford the allene **6a** (0,47 g, 83%) as a colorless oil. R_f = 0.74 (isohexane/EtOAc 85:15); $[\alpha]^{20}_D$ –41.3 (*c* 1.05, $CHCl_3$); ^{1}H NMR (400 MHz, $CDCl_3$): δ = 7.34 (m, 5H), 5.31–5.04 (m, 3H), 4.46 (m, 1H), 4.08–4.02 (m, 3H), 3.90 (dd, J = 8.7 Hz, 1H), 1.98 (br d, 2H), 1.63, 1.53, 1.47 (3 s, 6H), 1.35–1.26 (m, 4H), 0.89–0.85 (m, 12H), 0.04 (s, 6H); ^{13}C NMR (100 MHz, $CDCl_3$): δ = 199.8 (C=*C*=C), 152.2 (N*C*O_2CH_2Ph), 136.7 (Cq, arom.), 128.4, 128.0, 127.8, 127.5 (4 CH, arom.), 107.7 (HC=C=*C*q), 94.4 [*C*$(CH_3)_2$], 93.4, 92.5 (2 H*C*=C=Cq), 68.7, 67.9 (2 CH_2), 67.0, 66.3 (2 CH_2), 64.0 (CH_2), 56.8, 56.2 (2 CHN), 29.5 (CH_2), 28.6 (2 CH_2), 27.2, 26.3 [2 C(*C*$H_3)_2$], 25.8 [SiC(*C*$H_3)_3$], 25.0, 23.6 [2 C(*C*$H_3)_2$], 22.4 (CH_2), 18.1 [Si*C*$(CH_3)_3$], 13.9 (CH_3), –5.3 [Si$(CH_3)_2$]; IR (film): 3067, 3034, 2956, 2930, 2858, 1968 (C=C=C), 1708, 1498, 1463, 1404, 1384, 1350, 1254, 1209, 1145, 1075, 838, 776, 697 cm^{-1}; HRMS (ESI): *m/z* [*M* + H]$^+$ calcd for $C_{27}H_{44}NO_4{}^{28}Si$: 474.3034; found: 474.3035.

(*R*,*R*)-3-Benzyloxycarbonyl-4-(3'-bromo-4'-(*tert*-butyldimethylsilyloxy)buta-1',2'-dienyl)-2,2-dimethyloxazolidine (6b). Anhydrous LiBr (443 mg, 5.10 mmol, 6.0 equiv) was transferred into a 100 mL flask equipped with a magnetic stirring bar and nitrogen inlet. The flask was evacuated and heated with a heating gun to ~300 °C for several minutes. After cooling, the flask was flushed slowly with argon and CuBr·SMe_2 (1.05 g, 5.10 mmol, 6.0 equiv) and anhydrous THF (25 mL) were added. The suspension was stirred for 20 min at rt and a solution of tosylate **5a** (500 mg, 0.85 mmol, 1.0 equiv) in anhydrous THF (5 mL) was added via syringe. A condenser was put on the flask and the suspension was heated to 65 °C (bath temperature) giving a clear solution. After 6 h, the solution was cooled to rt and stirred overnight at

rt. It was heated to reflux for further 3 h, after which TLC control indicated complete conversion of the starting material. The solution was cooled to rt and was quenched by addition of aq sat. NH_4Cl (50 mL). After dilution with water, the organic phase was separated and the residue was extracted with diethyl ether (2 × 30 mL). The combined organic layers were washed with brine, dried ($MgSO_4$), filtered and the solvent was evaporated. The residue was purified by column chromatography (silica gel, isohexane/EtOAc 100 → 85:15) to give the faster eluted S_N2 product (41 mg, 10%) following by the allene **6b** (285 mg, 68%) as a yellow oil. R_f = 0.55 (isohexane/EtOAc 85:15); $[\alpha]^{22}_D$ –88.5 (*c* 1.00, $CHCl_3$); ^1H NMR (400 MHz, $CDCl_3$): δ = 7.35 (m, 5H, 5.50 (m, 1H, 5.27–5.06 (m, 2H, 4.58 (m, 1H, 4.23 (m, 2H, 4.09–4.05 (dd, 1H, 3.92 (d, 1H, 1.66, 1.60, 1.54, 1.48 (4 s, 6H, 0.90 (m, 9H, 0.08 (s, 6H; ^{13}C NMR (100 MHz, $CDCl_3$): δ = 198.5 (C=*C*=C), 151.9 (NCO_2Bn), 136.5, 136.1 (2 Cq, arom.), 128.4, 128.0, 127.9 (3 CH, arom.), 100.1, 99.3 (2 H*C*=C=Cq), 95.0, 94.6, 94.5, 94.2 [2 $C(CH_3)_2$, 2 HC=C=*C*q], 68.1, 67.4, 67.1, 66.6, 65.5 (2 CH_2), 55.8, 55.5 (2 CHN), 27.2, 26.3 [2 $C(CH_3)_2$], 25.7 [$SiC(CH_3)_3$], 24.8, 23.5 [2 $C(CH_3)_2$], 18.3 [$SiC(CH_3)_3$], –5.3 (2 C) [2 $Si(CH_3)_2$]; IR (film): 3091, 3066, 3033, 2983, 2954, 2930, 2884, 2857, 1971 (C=C=C), 1709, 1498, 1471, 1463, 1404, 1384, 1365, 1349, 1256, 1209, 1149, 1096, 1055, 1030, 1006, 837, 778, 697 cm^{-1}; HRMS (ESI): *m/z* $[M + H]^+$ calcd for $C_{23}H_{35}O_4N^{81}Br^{28}Si$: 498.1493; found: 498.1487.

(*R*,*R*)-3-Benzyloxycarbonyl-4-(4’-(*tert*-butyldimethylsilyloxy)-3’-(dimethylphenyl-silanyl)-1’,2’-butadienyl)-2,2-dimethyloxazolidine (6c). In an oven-dried Schlenk tube equipped with a magnetic stirring bar lithium chippings (107 mg, 15.4 mmol) in dry THF (3.5 mL) were cooled to 0 °C (bath temperature). Dimethylphenylsilyl chloride (0.51 mL, 3.1 mmol) was added dropwise. After a few min, the suspension turned red, and stirring at 0 °C was continued overnight. 1.2 mL (1.02 mmol) of the

supernatant $Me_2PhSiLi$ solution were added by syringe to a suspension of CuCN (46 mg, 0.51 mmol) in dry THF (6 mL) which was cooled to 0 °C. The resulting dark solution was stirred at 0 °C for 30 min and then cooled to –78 °C. A solution of tosylate **5a** (0.25 g, 0.43 mmol) in anhydrous THF (1.5 mL) was added over 20 min by syringe on the inner surface of the flask. After stirring for 10 min, TLC control indicated complete conversion of the starting material. The reaction was quenched by addition of aq sat. NH_4Cl (2 mL) and the cooling bath was removed. After stirring at rt for 30 min, isohexane/diethyl ether (1:1, 50 mL) was added, and the mixture was stirred for 15 min. The supernatant solution was filtered, and the residue was treated again with isohexane/diethyl ether (1:1, 30 mL). The combined filtrates were concentrated, and the residue was purified by column chromatography (silica gel, isohexane/EtOAc 93:7) to afford the allene **6c** (0,18 g, 77%) as a colorless oil. R_f = 0.64 (isohexane/EtOAc 85:15); $[\alpha]^{20}_D$ –67.0 (*c* 1.00, $CHCl_3$); ^1H NMR (400 MHz, $CDCl_3$): δ = 7.34 (m, 10H, 5.18 (m, 3H, 4.48 (m, 1H, 4.19 (s, 2H, 3.99 (dd, 1H, 3.88 (br d, 1H, 1.67–1.47 (m, 6H, 0.84 (s, 9H, 0.40 (m, 6H, –0.03 (d, 6H; ^{13}C NMR (100 MHz, $CDCl_3$): δ = 204.5, 204.2 (2 C=*C*=C), 152.4, 152.1 (2 N*C*O_2CH_2Ph), 137.5, 136.7 (2 Cq, arom.), 134.2, 133.8 (2 CH, arom.), 129.0, 128.4, 127.9, 127.8, 127.6 (5 CH, arom.), 127.3 (Cq, arom.), 100.5 (HC=C=*C*q), 94.4, 93.8 [2 *C*$(CH_3)_2$], 88.4, 87.4 (2 H*C*=C=Cq), 68.6, 67.9 (2 CH_2), 67.0, 66.3 (2 CH_2), 63.0 (CH_2), 56.3, 55.2 (2 CHN), 27.3, 26.3 [2 C($C$$H_3$)$_2$], 25.8 [SiC(*C*$H_3$)$_3$], 25.1, 23.7 [2 C(*C*$H_3$)$_2$], 18.3 [Si*C*$(CH_3)_3$], –2.6, –5.5 [2 $Si(CH_3)_2$]; IR (film): 3068, 3048, 3033, 2982, 2955, 2931, 2884, 2857, 1941 (C=C=C), 1705, 1463, 1404, 1384, 1350, 1253, 1209, 1142, 1094, 1067, 836, 816, 778, 734, 699 cm^{-1}; HRMS (ESI): *m/z* $[M + H]^+$ calcd for $C_{31}H_{46}NO_4{}^{28}Si_2$: 552.2960; found: 552.2959.

(4*R*,1'*S*)-3-Benzyloxycarbonyl-4-(3'-hydroxymethylhepta-1',2'-dienyl)-2,2-dimethyloxazolidine (7a). In a round bottom flask, the allene **6a** (1.12 g, 2.36 mmol) was dissolved in THF (24 mL). After cooling to 0 °C, *n*-Bu_4NF·3 H_2O (1.19 g, 3.55 mmol) was added in one portion and the mixture was stirred at 0 °C for 12 h. The reaction was quenched by addition of aq sat. NH_4Cl (25 mL), the organic phase was separated and the residue was washed with diethyl ether (4 × 30 mL). The combined organic layers were washed with brine, dried ($MgSO_4$), filtered and the solvent was evaporated. The residue was purified by column chromatography (silica gel, isohexane/EtOAc 7:3) to give the title compound **7a** (0.66 g, 78%) as a colorless oil. $[\alpha]^{20}_{D}$ –123.7 (*c* 0.98, $CHCl_3$); R_f = 0.35 (isohexane/EtOAc 7:3); ^{1}H NMR (400 MHz, $CDCl_3$): δ = 7.34 (m, 5H, 5.40 (d, 1H, 5.19 (m, 1H, 5.08 (m, 1H, 4.51 (d, 1H, 4.07–3.62 (m, 4 Hz), 1.96–1.75 (m, 2H, 1.63, 1.55, 1.52, 1.48 (4 s, 6H, 1.40–1.26 (m, 4H, 0.86 (t, *J* = 7.2 Hz, 3H; ^{13}C NMR (100 MHz, $CDCl_3$): δ = 198.2, 197.2 (2 C=*C*=C), 153.2, 152.0 (2 N*C*O_2CH_2Ph), 136.4, 136.1 (2 Cq, arom.), 128.5, 128.4, 128.1, 128.0, 127.7 (CH, arom.), 110.7, 109.8 (HC=C=*C*q), 95.3, 95.2 (H*C*=C=Cq), 94.6, 94.2 [2 *C*$(CH_3)_2$], 67.7, 67.5, 67.2, 66.6 (4 CH_2), 62.8, 61.8 (2 CH_2), 56.4, 55.7 (2 CHN), 29.7 (CH_2), 28.9 (CH_2), 26.9, 26.1, 24.6, 23.5 [4 C($C$$H_3)_2$], 22.4 ($CH_2$), 13.8 ($CH_3$); IR (film): 3454 (br), 3063, 3033, 2955, 2928, 2871, 1965 (C=C=C), 1704, 1498, 1456, 1408, 1384, 1352, 1254, 1208, 1143, 1095, 1054, 1029, 830, 764, 738, 698 cm^{-1}; HRMS (ESI): *m/z* $[M + H]^+$ calcd for $C_{21}H_{30}NO_4$: 360.2169; found: 360.2175.

(*R*,*R*)-3-Benzyloxycarbonyl-4-(3'-bromo-4'-hydroxybuta-1',2'-dienyl)-2,2-dimethyloxazolidine (7b). In a round bottom flask, the allene **6b** (250 mg, 0.50 mmol) was dissolved in THF (5 mL). After cooling to –45 °C, *n*-Bu_4NF·3 H_2O (237 mg, 0.75 mmol) was added in one portion and the mixture was allowed to warm to –5 °C over 2 h. The reaction was quenched by addition of aq sat. NH_4Cl (20 mL).

After dilution with water (~5 mL) and diethyl ether (20 mL), the organic phase was separated and the residue was washed with diethyl ether (4 × 20 mL). The combined organic layers were washed with brine, dried ($MgSO_4$), filtered and the solvent was evaporated. The residue was purified by column chromatography (silica gel, isohexane/EtOAc 7:3 → 1:1) to give the title compound **7b** (166 mg, 87%) as a colorless oil. $[\alpha]^{20}_D$ –212.9 (*c* 0.98, $CHCl_3$); R_f = 0.45 (isohexane/EtOAc 3:2); ^1H NMR (400 MHz, $CDCl_3$): δ = 7.36 (m, 5H, 5.54 (br m, 1H, 5.27–5.07 (m, 2H, 4.60 (br m, 1H, 4.16–3.92 (m, 4H, 3.58 (dd, 1H, 1.66 (br s, 3H, 1.52 (d, 3H; ^{13}C NMR (100 MHz, $CDCl_3$): δ = 197.8, 196.7 (2 C=*C*=C), 153.4, 151.8 (2 NCO_2Bn), 136.2, 135.9 (2 Cq, arom.), 128.7, 128.5, 128.2, 128.1, 127.9, 127.7 (6 CH, arom.), 102.2, 100.7 (2 H*C*=C=Cq), 95.1 (HC=C=*C*q), 94.8, 94.7 [2 *C*$(CH_3)_2$], 67.4, 67.2, 66.8, 65.0, 64.0 (5 CH_2), 56.1, 55.2 (2 CHN), 27.1, 26.2, 24.4, 23.4 [4 C$(CH_3)_2$]; IR (film): 3432 (br), 3093, 3064, 3031, 2985, 2937, 2877, 1973 (C=C=C), 1702, 1498, 1455, 1411, 1384, 1352, 1255, 1209, 1150, 1097, 1068, 1053, 1029, 929, 750, 698, 600 cm^{-1}; HRMS (ESI): *m/z* $[M + H]^+$ calcd for $C_{17}H_{21}NO_4{}^{79}Br$: 382.0649; found: 382.0652.

(4*R*,2'*S*)-3-Benzyloxycarbonyl-4-(3'-aminomethylhepta-1',2'-dienyl)-2,2-dimethyloxazolidine (8a). In an oven-dried Schlenk tube equipped with a magnetic stirring bar, α-hydroxyallene **7a** (1.00 g, 2.78 mmol), phthalimide (0.82 g, 5.56 mmol) and PPh_3 (1.46 g, 5.56 mmol) were dissolved in anhydrous THF (18 mL) under argon. The mixture was cooled to 0 °C and DEAD (0.90 mL, 5.56 mmol) was slowly added dropwise over 5 min. After 20 min with stirring, TLC control showed complete conversion of the starting material. After 45 min total time of stirring, isohexane (50 mL) was added rapidly. A precipitate was formed and stirring was continued for 15 min at rt. The supernatant solution was filtered and the filtrate was concentrated under reduced pressure. The residue was purified by column chromatography (silica

gel, isohexane/EtOAc 4:1 → 7:3) to give the phthalimide intermediate (1.36 g) as a colorless oil. In a 100 mL round-bottom flask equipped with magnetic stirring bar and reflux condenser, the phthalimide intermediate was dissolved in EtOH (28 mL) and $N_2H_4{\cdot}H_2O$ (0.27 mL, 5.56 mmol) was added. The mixture was heated to reflux for 1 h, after which a white precipitate was formed. After cooling, EtOAc (40 mL) was added and the suspension was stirred vigorously for 20 min at rt. The suspension was filtered with suction and the residue was washed with a small amount of EtOAc. The filtrate was concentrated under reduced pressure and the residue was purified by column chromatography (CH_2Cl_2/MeOH 10:1) to yield the title compound **8a** (0.67 g, 67%) as a yellow oil. $[\alpha]^{22}_{D}$ –109.2 (*c* 1.00, $CHCl_3$); R_f = 0.28 (CH_2Cl_2/MeOH 10:1); ^{1}H NMR (400 MHz, $CDCl_3$): δ = 7.36 (m, 5H, 5.45 (d, 1H, 5.20 (d, 1H, 5.08 (d, 1H, 4.51 (br d, 1H, 4.02 (m, 1H, 3.92 (m, 1H, 3.13 (m, 1 Hz), 1.89 (m, 4H, 1.62 / 1.56 / 1.53 / 1.49 (4 s, 6H, 1.31 (m, 4H, 0.87 (t, 3H; ^{13}C NMR (100 MHz, $CDCl_3$): δ = 198.1, 197.1 (2 C=*C*=C), 153.0, 152.0 (2 NCO_2Bn), 136.4, 136.2 (Cq, arom.), 128.7, 128.3, 128.2, 128.1, 127.8, 127.6 (CH, arom.), 111.0, 108.3 (HC=C=*C*q), 96.8, 95.5 (H*C*=C=Cq), 94.4, 94.1 [2 *C*(CH_3)$_2$], 67.7, 67.5, 67.2, 66.4 (4 CH_2), 56.3, 55.9 (2 CHN), 43.3, 41.0 (2 CH_2), 30.0, 29.9 (2 CH_2), 29.6, 29.4 (2 CH_2), 27.0, 26.2, 24.4, 24.0 [4 C(*C*H_3)$_2$], 22.3 (CH_2), 13.7 (CH_3); IR (film): 3371 (br), 3091, 3064, 3033, 2982, 2956, 2932, 2872, 1965 (C=C=C), 1703, 1456, 1407, 1383, 1352, 1255, 1208, 1143, 1094, 1069, 1055, 842, 829, 751, 698 cm^{-1}; HRMS (ESI): *m/z* [*M* + H]$^+$ calcd for $C_{21}H_{31}N_2O_3$: 359.2329; found: 359.2328.

(*R*,*R*)-3-Benzyloxycarbonyl-4-(4’-amino-3’-bromobuta-1’,2’-dienyl)-2,2-dimethyl-oxazolidine (8b). Similar to the synthesis of **8a**, α-hydroxyallene **7b** (460 mg, 1.20 mmol), PPh_3 (629 mg, 2.40 mmol) and phthalimide (353 mg, 2.40 mmol) were stirred with DEAD (40% in toluene, 1.05 mL, 418 mg, 2.40 mmol) in anhydrous THF (8 mL)

for 30 min at 0 °C. Work-up as described above gave the phthalimide intermediate (600 mg), which was refluxed with $N_2H_4 \cdot H_2O$ (0.29 mL, 6.00 mmol) in EtOH (12 mL). After dilution with EtOAc and removal of the solvent, column chromatography (CH_2Cl_2/MeOH 10:1) gave the title compound **8b** (225 mg, 49% over two steps) as a yellow oil. $[\alpha]^{21}_{D}$ –151.2 (*c* 1.00, $CHCl_3$); R_f = 0.54 (CH_2Cl_2/MeOH 10:1); ^1H NMR (400 MHz, $CDCl_3$): δ = 7.32 (m, 5H, 5.47 (m, 1H, 5.22–5.03 (m, 2H, 4.55 (m, 1H, 4.03 (dd, 1H, 3.90 (m, 1H, 3.36 (br s, 1H, 3.17 (dd, 1H, 1.62 (m, 5H, 1.50, 1.47 (2 s, 3H; ^{13}C NMR (100 MHz, $CDCl_3$): δ = 197.1, 196.9 (2 C=*C*=C), 152.5, 151.7 (2 NCO_2Bn), 136.1, 136.1 (2 Cq, arom.), 128.5, 128.4, 128.3, 128.1, 128.0, 127.8, 127.6 (7 CH, arom.), 101.4, 100.5 (2 H*C*=C=Cq), 98.2 (HC=C=*C*q), 94.6, 94.3 [2 *C*$(CH_3)_2$], 67.3, 67.0, 66.6 (3 CH_2), 55.8, 55.1 (2 CHN), 48.0, 47.6 (2 CH_2), 27.1, 26.2, 24.4, 23.3 [4 C(CH_3)$_2$]; IR (film): 3373 (br), 3090, 3064, 3032, 2984, 2973, 2878, 1971 (C=C=C), 1699, 1498, 1456, 1408, 1384, 1352, 1256, 1208, 1148, 1094, 1068, 1054, 1029, 968, 912, 841, 823, 764, 749, 698, 599 cm^{-1}; HRMS (ESI): *m/z* $[M + H]^+$ calcd for $C_{17}H_{22}N_2O_3{}^{79}Br$: 381.0808; found: 381.0816.

(4*S*,2'*R*)-3-Benzyloxycarbonyl-4-(4'-butyl-2',5'-dihydrofuran-2'-yl)-2,2-dimethyloxazolidine (9a). In an oven-dried Schlenk tube equipped with a magnetic stirring bar, α-hydroxyallene **7a** (50 mg, 0.14 mmol) was dissolved in anhydrous THF (1 mL). After cooling to 0 °C, $AuCl_3$ (0.20 M in acetonitrile, 7.0 µL, 1 mol %) was added via syringe and the yellow solution was stirred for 14 h at 5 °C, after which TLC control showed complete conversion of the starting material. The reaction was quenched with aq sat. $NaHCO_3$ (3 mL) and the mixture was extracted with diethyl ether (3 × 3 mL). The combined organic extracts was washed with brine, dried ($MgSO_4$), filtered and the solvent was evaporated. The residue was purified by column chromatography (silica gel, isohexane/EtOAc 93:7) to give the title compound

9a (42 mg, 84% yield) as a colorless oil. $[\alpha]^{25}_{D}$ –1.9 (*c* 0.59, $CHCl_3$), R_f = 0.56 (isohexane/EtOAc 85:15); ^1H NMR (400 MHz, $CDCl_3$): δ = 7.34 (m, 5H, 5.37, 5.29 (2 s, 1H, 5.14 (d, 2H, 5.08, 4.91 (2 s, 1H, 4.51 (m, 2H, 4.00–3.86 (m, 3H, 2.08 (m, 1H, 1.96 (m, 1H, 1.64–1.23 (m, 10H, 0.89 (m, 3H; ^{13}C NMR (100 MHz, $CDCl_3$): δ = 153.2, 152.5 (2 NCO_2Bn), 142.3 (HC=*C*q), 136.3, 136.2 (Cq, arom.), 128.4, 128.0, 127.9 (3 CH, arom.), 120.1 (H*C*=Cq), 94.7, 94.2 [2 *C*(CH_3)$_2$], 86.4, 85.8 (2 CH), 77.3, 77.1 (2 CH_2), 67.1, 66.6 (2 CH_2), 64.9, 64.0 (2 CH_2), 61.3, 60.7 (2 CHN), 29.6 (CH_2), 26.9 [C(*C*H_3)$_2$], 26.8 (2 CH_2), 26.3, 24.9, 23.2 [3 C(*C*H_3)$_2$], 22.4 (CH_2), 13.8 (CH_3); IR (film): 3062, 3033, 2956, 2930, 2873, 1702, 1456, 1406, 1384, 1351, 1254, 1209, 1151, 1075, 1052, 1029, 928, 765, 698 cm^{-1}; HRMS (ESI): *m/z* [*M* + H]$^+$ calcd for $C_{21}H_{30}NO_4$: 360.2169; found: 360.2175.

(4*S*,2’*R*)-3-Benzyloxycarbonyl-4-(4’-brom-2’,5’-dihydrofuran-2’-yl)-2,2-dimethyloxazolidine (9b). In an oven-dried Schlenk tube equipped with a magnetic stirring bar, a solution of the α-hydroxyallene **7b** (111 mg, 0.29 mmol) and $AuCl_3$ (0.20 M in acetonitrile, 29.0 µL, 1 mol %) in anhydrous THF (2 mL) was stirred for 5 h at 50 °C. After cooling, the solvent was removed under reduced pressure and the residue was purified by column chromatography (silica gel, isohexane/EtOAc 85:15) to give the title compound **9b** as an inseparable mixture of diastereoisomers (79 mg, 77% yield, *dr* = 4:1, by NMR analysis). Colorless oil; R_f = 0.87 (isohexane/EtOAc 7:3); ^1H NMR (400 MHz, $CDCl_3$): δ = 7.36 (m, 5H, 5.98–5.86 (m, 1H, 5.19–5.05 (m, 2H, 4.96, 4.80 (2 m, 1H, 4.56 (m, 2H, 4.04–3.89 (m, 2H, 1.65–1.46 (m, 6H; ^{13}C NMR (100 MHz, $CDCl_3$): δ = 153.3, 152.3 (2 NCO_2Bn), 136.0 (Cq, arom.), 128.6, 128.6, 128.4, 128.2, 128.1, 128.0, 127.8 [7 CH, arom. / (H*C*=Cq)], 116.6, 116.4 (2 HC=*C*q), 94.8, 94.4 [2 *C*(CH_3)$_2$], 86.4, 86.2 (2 CH), 78.2, 78.1, 78.0 (3 CH_2), 67.3, 67.0, 66.8 (3 CH_2), 64.9, 64.0, 63.7, 63.4 (4 CH_2), 60.8, 60.2, 59.9, 59.1 (4 CHN), 27.1, 26.5, 26.1,

24.6, 24.0, 23.0, 22.6 [7 C(CH_3)$_2$]; IR (film): 3091, 3065, 3033, 2984, 2939, 2879, 1781, 1705, 1627, 1498, 1456, 1406, 1380, 1351, 1300, 1256, 1210, 1153, 1085, 1051, 1028, 915, 861, 838, 765, 699 cm^{-1}; HRMS (ESI): *m/z* [*M* + H]$^+$ calcd for $C_{17}H_{21}{}^{79}BrNO_4$: 382.0649; found: 382.0651.

(*R*,*R*)-3-Benzyloxycarbonyl-4-(4'-butyl-2',5'-dihydro-1*H*-pyrrol-2-yl)-2,2-dimethyloxazolidine (10a). A solution of α-aminoallene **8a** (124 mg, 0.35 mmol), and imidazole (2.4 mg, 0.04 mmol, 10 mol %) in toluene (2.3 mL) was treated with AuCl (8.0 mg, 0.04 mmol, 10 mol %), and the mixture was stirred for 12 h at 80 °C. After cooling to rt, the mixture was concentrated under reduced pressure and the residue was purified by column chromatography (silica gel, CH_2Cl_2/MeOH 10:1) to give 97 mg of title compound **10a** (78% yield) as orange oil. $[\alpha]^{20}_D$ –1.7 (*c* 1.00, $CHCl_3$); R_f = 0.32 (CH_2Cl_2/MeOH 10:1); ^1H NMR (400 MHz, $CDCl_3$): δ = 7.34 (m, 5H, 5.30–5.09 (m, 3H, 4.78 (br s, 1H, 4.63, 4.31 (2 s, 2H, 4.06–3.76 (m, 3H, 3.63–3.51 (m, 1 Hz), 2.00 (m, 2H, 1.64, 1.53, 1.45 (3 s, 6H, 1.31 (ddd, 4H, 0.88 (m, 3H; ^{13}C NMR (100 MHz, $CDCl_3$): δ = 154.9, 152.6 (2 N*C*O_2CH_2Ph), 144.2, 143.7 (2 HC=*C*q), 136.3, 135.7 (2 Cq, arom.), 128.6, 128.5, 128.4, 128.2, 128.0, 127.9 (6 CH, arom.), 121.6, 119.2 (H*C*=Cq), 95.0, 94.8 [2 *C*(CH_3)$_2$], 69.1 (CH), 67.9 (CH_2), 67.6 (CH), 66.6, 65.3 (2 CH_2), 55.9, 54.5 (2 CH_2), 29.7, 29.4 (2 CH_2), 28.4, 28.2 (2 CH_2), 26.3, 26.2, 24.1, 22.9 [4 C(CH_3)$_2$], 22.5, 22.4 (2 CH_2), 13.8 (CH_3); IR (film): 3373, 3090, 3064, 3033, 2980, 2956, 2932, 2872, 2860, 1703, 1498, 1456, 1406, 1380, 1351, 1254, 1207, 1148, 1094, 1071, 1055, 1029, 836, 765, 750, 698, 602 cm^{-1}; HRMS (ESI): *m/z* [*M* + H]$^+$ calcd for $C_{21}H_{31}N_2O_3$: 359.2329; found: 359.2329.

(*R*,*R*)-3-Benzyloxycarbonyl-4-(4'-bromo-2',5'-dihydro-1*H*-pyrrol-2-yl)-2,2-dimethyloxazolidine (10b). Similar to the synthesis of dihydropyrrole **10a**, a solution

of α-aminoallene **8b** (215 mg, 0.56 mmol), Ph_3PAuCl (19.8 mg, 0.04 mmol, 7 mol %) $AgBF_4$ (7.8 mg, 0.04 mmol, 7 mol %) and imidazole (2.7 mg, 0.04 mmol, 7 mol %) in toluene (2.3 mL) was stirred for 16 h at 100 °C. After cooling to rt, the mixture was concentrated under reduced pressure and the residue was purified by column chromatography (silica gel, CH_2Cl_2/MeOH 10:1) to give 180 mg of a brown oil contaminated with ~10% of impurities (NMR analysis). $[\alpha]^{20}_D$ –1.4 (*c* 1.00, $CHCl_3$); R_f = 0.64 (CH_2Cl_2/MeOH 10:1); ^{1}H NMR (400 MHz, $CDCl_3$): δ = 7.35 (m, 5H, 5.84 (d, 1H, 5.11 (m, 2H, 4.33–3.77 (m, 6H, 2.27 (br s, 1H, 1.63, 1.56, 1.53, 1.44 (4 s, 6H; ^{13}C NMR (100 MHz, $CDCl_3$): δ = 153.9, 152.4 (2 NCO_2Bn), 136.0 (Cq, arom.), 128.5, 128.2, 128.1, 127.9 (4 CH, arom., H*C*=Cq), 119.8, 119.6 (2 HC=*C*q), 94.8, 94.6 [2 *C*(CH_3)$_2$], 68.3, 67.6 (2 CH), 67.3, 66.9 (2 CH_2), 65.3, 64.7 (2 CH_2), 60.8, 60.5 (2 CHN), 58.8, 58.8 (2 CH_2), 26.7, 26.4, 24.4, 22.8 [4 C(*C*H_3)$_2$]; IR (film): 3365, 3090, 3065, 3032, 2983, 2938, 2878, 1698, 1624, 1498, 1455, 1406, 1380, 1351, 1286, 1255, 1208, 1148, 1092, 1073, 1048, 1029, 861, 828, 766, 750, 698 cm^{-1}; HRMS (ESI): *m/z* [*M* + H]$^+$ calcd for $C_{17}H_{21}N_2O_3{}^{79}Br$: 381.0808; found: 381.0816.

(*R*,*R*)-3-Benzyloxycarbonyl-4-[1'-benzyloxycarbonyl-4'-butyl-2',5'-dihydro-1*H*-pyrrol-2-yl]-2,2-dimethyloxazolidine (11a). In a 50 mL round bottom flask with magnetic stirring bar, to a vigorously stirred solution of dihydropyrrole **10a** (120 mg, 0.34 mmol) und DMAP (117 mg, 0.96 mmol, 2.85 equiv) in acetonitrile (6.5 mL) was added CbzCl (136 µL, 0.96 mmol, 2.85 equiv) at rt. After 30 min, the conversion was complete as indicated by TLC. After 40 min total time of stirring, aq sat. NH_4Cl (30 mL) was added and the mixture was extracted with diethyl ether (4 × 30 mL). The combined organic extract was washed with brine, dried ($MgSO_4$), filtered and the solvent was evaporated. The residue was purified by column chromatography (silica gel, isohexane/EtOAc 4:1) to give the title compound **10a** (130 mg, 79%) as a yellow

oil. $[\alpha]^{20}_{D}$ +43.6 (*c* 1.00, $CHCl_3$); R_f = 0.40 (isohexane/EtOAc 4:1); ^{1}H NMR (400 MHz, $CDCl_3$): δ = 7.35 (m, 10H, 5.35 (m, 1H, 5.27–5.00 (m, 2H, 4.90–4.68 (m, 3H, 4.48–3.76 (m, 5H, 2.06 (m, 2H, 1.58 (s, 2H, 1.48–1.26 (m, 8H, 0.90 (t, 3H; ^{13}C NMR (100 MHz, $CDCl_3$): δ = 154.4, 153.4, 153.1, 152.9 (4 NCO_2Bn), 142.0, 141.6, 141.4 (3 HC=*C*q), 136.6, 136.5, 136.5, 136.4 (4 CH, arom.), 128.2, 128.1, 128.0, 127.7, 127.6, 127.5 (CH, arom.), 119.4 (H*C*=Cq), 95.2 (2 C), 94.6, 94.5 [4 *C*$(CH_3)_2$], 68.5, 67.7, 66.9 (3 CH), 66.7, 66.6, 66.4, 66.3, 65.3, 65.2 (6 CH_2), 59.5, 58.6, 58.3, 57.9 (4 CHN), 56.0, 55.3 (2 CH_2), 29.2 (CH_2), 28.4 (CH_2), 25.9, 25.0, 24.4, 24.3, 22.8, 22.7 [6 C($C$$H_3$)$_2$], 22.3 ($CH_2$), 13.7 ($CH_3$); IR (film): 3089, 3064, 3033, 2956, 2932, 2873, 1702, 1660, 1498, 1455, 1418, 1384, 1351, 1256, 1235, 1205, 1098, 1071, 1058, 1029, 764, 698 cm^{-1}; HRMS (ESI): *m/z* [*M* + H]$^+$ calcd for $C_{29}H_{37}N_2O_5$: 493.2697; found: 493.2691.

(*R*,*R*)-3-Benzyloxycarbonyl-4-(1'-benzyloxycarbonyl-4'-bromo-2',5'-dihydro-1*H*-pyrrol-2-yl)-2,2-dimethyloxazolidine (11b). Similar to the preparation of **11a**, a vigorously stirred solution of crude dihydropyrrole **9b** (180 mg, 0.47 mmol) and DMAP (164 mg, 1.34 mmol, 2.85 equiv) in acetonitrile (10 mL) was treated with CbzCl (191 µL, 1.34 mmol, 2.85 equiv) at rt. After 40 min total time of stirring, aq sat. NH_4Cl (40 mL) was added. After dilution with water (~5 mL), the organic phase was separated and the residue was extracted with diethyl ether (4 × 40 mL). The combined organic layers were washed with brine, dried ($MgSO_4$), filtered, and the solvent was evaporated. The residue was purified by column chromatography (silica gel, isohexane/EtOAc 4:1) to give the epimer (4*R*,2'*S*)-**11b** (10 mg, 4% over two steps) and the title compound **11b** (130 mg, 47% yield over two steps starting from **8b**) as a colorless oil. $[\alpha]^{20}_{D}$ +26.5 (*c* 1.07, $CHCl_3$); R_f = 0.43 (isohexane/EtOAc 7:3); ^{1}H NMR (400 MHz, $CDCl_3$): δ = 7.32 (m, 10H, 5.92 (m, 1H, 5.36–5.03 (m, 3H, 4.82–

4.64 (m, 3H, 4.46–3.75 (m, 5H, 1.74–1.42 (m, 6H; ^{13}C NMR (100 MHz, $CDCl_3$): δ = 153.9, 153.7, 153.2, 153.1 (4 NCO_2Bn), 136.4, 136.2, 136.1 (3 Cq, arom.), 128.5, 128.4, 128.2, 128.1, 127.9, 127.7, 127.5, 127.2 (8 CH, arom., H*C*=Cq), 116.9, 116.3, 116.0 (3 HC=*C*q), 95.7, 95.7 [2 *C*(CH_3)$_2$], 69.1, 68.5, 67.9 (3 CH), 67.2, 67.1, 66.9, 66.7, 66.1, 65.4, 65.3 (7 CH_2), 58.2 (CH_2), 57.7 (CHN), 57.6, 57.4 (2 CH_2), 26.2, 25.4, 24.3, 22.8 [4 C(*C*H_3)$_2$]; IR (film): 3090, 3064, 3033, 2982, 2941, 2881, 1705, 1631, 1498, 1455, 1416, 1382, 1347, 1256, 1211, 1151, 1117, 1094, 1073, 1055, 835, 763, 698 cm^{-1}; HRMS (ESI): *m/z* [*M* + H]$^+$ calcd for $C_{25}H_{28}N_2O_5{}^{79}Br$: 515.1176; found: 515.1173. Analytical data for the epimer (4*R*,2'*S*)-**11b**: colorless oil; $[\alpha]^{20}_D$ –47.0 (*c* 0.10, $CHCl_3$); R_f = 0.72 (isohexane/EtOAc 7:3); ^{1}H NMR (400 MHz, $CDCl_3$): δ = 7.49–7.32 (m, 10H, 5.90 (m, 1H, 5.24–4.70 (m, 6H, 4.39–4.18 (m, 2H, 3.94–3.82 (m, 1H, 3.73–3.61 (m, 1H, 1.64–1.41 (m, 6H; ^{13}C NMR (100 MHz, $CDCl_3$): δ = 154.3, 154.1, 152.8, 152.7 (4 NCO_2Bn), 136.3, 136.2 (2 Cq, arom.), 128.5, 128.3, 128.2, 128.1, 127.1, 126.8 (6 CH, arom., H*C*=Cq), 115.5, 114.9 (2 HC=*C*q), 95.1 [*C*(CH_3)$_2$], 67.3, 67.2 (2 CH), 66.8 (CH_2), 66.2, 65.8, 65.6 (3 CH), 59.0, 58.6 (2 CH_2), 58.0, 57.2 (2 CHN), 26.6, 25.8, 24.1, 22.6 [4 C(*C*H_3)$_2$]; IR (film): 3090, 3064, 3032, 2985, 2939, 2876, 1705, 1631, 1498, 1455, 1407, 1383, 1361, 1348, 1325, 1260, 1101, 1055, 839, 766, 750, 698 cm^{-1}; HRMS (ESI): *m/z* [*M* + H]$^+$ calcd for $C_{25}H_{27}N_2O_5{}^{79}BrNa$: 537.0996; found: 537.0996.

(*R*,*R*)-*N*-Benzyloxycarbonyl-2-(1'-benzyloxycarbonyl-4'-butyl-2',5'-dihydro-pyrrol-2-yl)glycine (12a). In a 25 mL round-bottom flask equipped with magnetic stirring bar, *p*-TsOH·H_2O (17 mg, 0.09 mmol) was added to a solution of dihydropyrrole **11a** (215 mg, 0.44 mmol) in MeOH (5 mL) and the mixture was allowed to stir at rt overnight (~12 h). Further *p*-TsOH·H_2O (8 mg, 0.04 mmol) was added and after an additional 5 h of stirring, TLC control showed complete

conversion of the starting material. The mixture was concentrated under reduced pressure and the residue was purified by column chromatography (silica gel, isohexane/EtOAc 3:2 → 2:3) to give the hydroxycarbamate (178 mg, 90% yield) as a colorless oil. $[\alpha]^{21}_{D}$ +60.6 (*c* 0.50, $CHCl_3$); R_f = 0.21 (isohexane/EtOAc 3:2); ^{1}H NMR (400 MHz, $CDCl_3$): δ = 7.35 (m, 10H, 6.12 (d, 1H, 5.43 (s, 1H, 5.17–5.06 (m, 4H, 4.68 (s, 1H, 4.18 (d, 1H, 3.97 (dd, 1H, 3.85 (m, 1H, 3.63 (ddd, 2H, 2.07 (m, 2H, 1.43 (m, 2H, 1.32 (m, 2H, 0.91 (t, 3H; ^{13}C NMR (100 MHz, $CDCl_3$): δ = 156.5, 156.1 (2 NCO_2Bn), 141.0 (HC=*C*q), 136.4, 136.1 (2 Cq, arom.), 128.4, 128.3, 128.0, 127.9, 127.8 (5 CH, arom.), 120.3 (H*C*=Cq), 67.3 (CH_2), 66.6 (CH_2), 66.0 (CH), 62.5 (CH_2), 56.4 (CHN), 55.8 (CH_2), 29.3 (CH_2), 28.2 (CH_2), 22.2 (CH_2), 13.7 (CH_3); IR (film): 3335, 3089, 3065, 3033, 2955, 2930, 2871, 2859, 1704, 1656, 1512, 1499, 1455, 1418, 1384, 1362, 1339, 1304, 1235, 1215, 1181, 1114, 1082, 1062, 1028, 737, 698, 605 cm^{-1}; HRMS (ESI): *m/z* $[M + H]^+$ calcd for $C_{26}H_{33}N_2O_5$: 453.2384; found: 453.2380.

A solution of the hydroxycarbamate (100 mg, 0.22 mmol) and Dess–Martin periodinane [7,8] (124 mg, 0.33 mmol) in anhydrous CH_2Cl_2 (2.2 mL) was stirred 70 min at rt under argon, whereby TLC control indicated complete conversion of the starting material. After 85 min total time of stirring, the mixture was diluted with diethyl ether (10 mL) and sat. aq $NaHCO_3$/sat. aq $Na_2S_2O_3$ (1:1, 10 mL). After a few minutes stirring at rt, the biphasic mixture came clear. The organic phase was separated and the aqueous layer was washed with diethyl ether (4 × 10 mL). The combined organic layers were washed with aq sat. $NaHCO_3$ (10 mL), brine and dried ($MgSO_4$). Filtration and evaporation of the solvent gave the crude aldehyde (100 mg) which was used in the next step without delay.

In a 50 mL round-bottom flask with a magnetic stirring bar, the crude aldehyde (90 mg) and resorcine (32 mg, 0.29 mmol) were dissolved in dioxane (19 mL). The flask

was sealed with a rubber septum and the solution was cooled to 12 °C (bath temperature). With vigorous stirring, a solution of $NaClO_2$ (87 mg, 0.77 mmol, 80%, technical grade) and $NaH_2PO_4 \cdot H_2O$ (106 mg, 0.77 mmol) in water (1 mL) was added dropwise by syringe over 20 min, after which TLC monitoring showed complete consumption of the starting material. After 40 min total time of stirring, the slightly yellow reaction mixture was poured in aq sat. $NaHCO_3$ (20 mL) and the biphasic mixture was concentrated to its half volume under reduced pressure. $CHCl_3$ (20 mL) was added and after cooling to 0 °C, 1 M HCl was added dropwise with stirring to adjust the pH to 2–3. The organic phase was separated, and the aqueous layer was extracted twice with $CHCl_3$. The combined organic layers were dried ($MgSO_4$), filtered and the solvent was evaporated to give the crude product, which was dissolved in $CHCl_3$ and put on a silica gel column. Elution with isohexane/EtOAc (1:1) removed resorcine and chlorinated byproducts. Further elution with isohexane/EtOAc/AcOH (8:2:1) gave the product which was coevaporated with toluene several times for removing AcOH to yield 65 mg (65%) of Cbz-protected amino acid **12a** as a colorless oil. $[\alpha]^{21}_{D}$ +63.7 (*c* 0.53, $CHCl_3$); R_f = 0.39 (isohexane/EtOAc/AcOH 8:2:1); ^{1}H NMR (400 MHz, $CDCl_3$): δ = 8.73 (br s, 1H, 7.44–7.30 (m, 10H, 5.32–4.99 (m, 7H, 4.17–3.89 (m, 2H, 2.07 (m, 2H, 1.34 (m, 4H, 0.87 (t, *J* = 7.1 Hz, 3H; ^{13}C NMR (100 MHz, $CDCl_3$): δ = 174.1, 173.8 (2 COOH), 156.3, 155.0, 154.3 (3 NCO_2Bn), 145.1, 143.8 (2 HC=*C*q), 136.3, 136.2, 136.0 (3 Cq, arom.), 128.5, 128.4, 128.4, 128.2, 128.1, 128.1, 128.0, 127.9, 127.9 (9 CH, arom.), 117.4, 116.5 (2 H*C*=Cq), 67.5, 67.3, 67.2, 66.9 (4 CH_2), 66.7, 65.6 (2 CH), 56.1 (CH_2), 56.0 (CH), 55.7 (CH_2), 55.6 (CH), 29.4 (CH_2), 28.5, 28.4 (2 CH_2), 22.3 (CH_2), 13.8 (CH_3); IR (film): 3433 (br), 3320 (br), 3090, 3065, 3033, 2956, 2931, 2860, 2609 (br), 1713, 1657, 1514, 1455, 1417, 1384, 1361, 1217, 1155, 1118, 1056, 1029, 982,

913, 847, 753, 698 cm^{-1}; HRMS (ESI): *m/z* [*M* + H]$^+$ calcd for $C_{26}H_{31}N_2O_6$: 467.2177; found: 467.2172.

(*R*,*R*)-(4-Butyl-2,5-dihydro-1*H*-pyrrol-2-yl)glycine (13a). In a 25 mL round-bottom flask, TFA (4.5 mL) was added to a mixture of Cbz-protected amino acid **12a** (45 mg, 0.10 mmol) and thioanisole (165 µL, 1.40 mmol) at 0 °C. The cooling bath was removed and the solution was stirred overnight at rt. Volatile components were removed under reduced pressure and the dark residue was purified by ion exchange chromatography (Amberlite IR-120). The crude product was further purified on silica gel (iPrOH/H_2O 3:1) to yield amino acid **13a** (6 mg, 31%) with a purity ≥ 95% (NMR analysis). Colorless solid, mp. 140 °C (dec); $[\alpha]^{20}_{D}$ +9 (*c* 0.15, H_2O); ^{1}H NMR (400 MHz, CD_3OD/D_2O 1:1): δ = 5.37 (s, 1H 3-H), 4.64 (s, 1H 2-H), 3.92 (q, *J* = 15.3 Hz, 2H 5-H), 3.53 (d, *J* = 5.3 Hz, 1H C*H*N), 2.17 (m, 2H, 1.47 (m, 2H, 1.33 (qd, *J* = 7.3, 14.4 Hz, 2H, 0.90 (t, *J* = 7.3 Hz, 3H; ^{13}C NMR (100 MHz, CD_3OD/D_2O 1:1): δ = 176.5 (COOH), 145.7 (HC=*C*q), 118.5 (H*C*=Cq), 68.3 (CH), 58.0 (CH), 55.0 (CH_2), 30.1 (CH_2), 28.7 (CH_2), 23.1 (CH_2), 14.2 (CH_3); IR (film): 3334 (br), 3179 (br), 3055, 2956, 2929, 2871, 2859, 1715, 1607, 1436, 1409, 1385, 1346, 1180, 1119, 721, 695, 618, 542 cm^{-1}; HRMS (ESI): *m/z* [*M* + H]$^+$ calcd for $C_{10}H_{19}N_2O_2$: 199.1441; found: 199.1440.

(*R*,*R*)-3-Benzyloxycarbonyl-4-[4-(*tert*-butyldimethylsilyloxy)-1-hydroxybut-2'-inyl]-2,2-dimethyloxazolidine (14). In an oven-dried three-necked 250 mL flask equipped with a magnetic stirring bar and nitrogen inlet, **3** [3] (3.88 g, 22.8 mmol, 2 equiv) was dissolved in anhydrous diethyl ether (55 mL) under argon. After cooling to –78 °C, *n*-BuLi (10.0 mL, 22.8 mmol, 2 equiv, 2.35 M in hexane) was added slowly dropwise via syringe and stirring was continued for 15 min at –78 °C. A solution of

$ZnBr_2$ in diethyl ether (35.0 mL, 35.0 mmol, 3 equiv) was added via syringe and the solution was allowed to stir at –78 °C for 15 min. (After addition of the zinc salt, a white precipitate was formed and mechanical stirring was hampered. This had no influence on the yield and stereoselectivity. After warming to rt overnight, the precipitate was dissolved to give a clear stirred solution). A solution of aldehyde (*R*)-**2** [4–6] (3.00 g, 11.4 mmol) in anhydrous diethyl ether (10 mL) was added via syringe. The mixture was allowed to warm to rt overnight (~12 h) and stirring was continued for 9 h at rt, after which TLC control indicated complete conversion of the starting material. The mixture was poured in aq sat. NH_4Cl (100 mL) and the organic phase was separated. The residue was diluted with water and was extracted with diethyl ether (3 × 50 mL). The combined organic layers were washed with brine, dried ($MgSO_4$), filtered, and the solvent was evaporated. The residue was purified by column chromatography (silica gel, isohexane/EtOAc 85:15 → 4:1) to give the title compound **14** (4.13 g, 84% yield) as a pale yellow oil (*syn*:*anti* > 95:5, by NMR analysis). $[\alpha]^{21}_{D}$ +14.3 (*c* 1.00, $CHCl_3$); R_f = 0.54 (isohexane/EtOAc 7:3); ^1H NMR (400 MHz, $CDCl_3$): δ = 7.34 (m, 5H, 5.15 (br m, 2H, 4.65 (m, 1H, 4.32 (m, 2H, 4.24–4.0 (m, 3H, 1.66 / 1.57 / 1.52 / 1.46 (4 s, 6H, 0.89 (s, 9H, 0.10 (s, 6H; ^{13}C NMR (100 MHz, $CDCl_3$): δ = 155.2, 152.3 (2 NCO_2Bn), 136.0, 135.5 (2 Cq, arom.), 128.5, 128.3, 128.2, 128.0 (4 CH, arom.), 95.2, 94.9 [2 $C(CH_3)_2$], 84.9, 84.5, 83.1, 82.8 (4 C≡C), 67.9, 66.8 (2 CH_2), 65.2 (CH), 65.1, 65.0, 64.9 (3 CH_2), 63.2, 62.3, 60.6 (3 CH), 51.6 (CH_2), 27.1, 26.1 [2 $C(CH_3)_2$], 25.7 [$SiC(CH_3)_3$], 24.3, 23.1 [2 $C(CH_3)_2$], 18.1 [$SiC(CH_3)_3$], –5.3 [$Si(CH_3)_2$]; IR (film): 3435 (br), 3091, 3066, 3034, 2954, 2931, 2885, 2858, 1698, 1498, 1471, 1463, 1409, 1382, 1352, 1257, 1210, 1143, 1093, 1018, 837, 816, 779, 736, 698 cm^{-1}; HRMS (ESI): *m/z* $[M + H]^+$ calcd for $C_{23}H_{36}NO_5{}^{28}Si$: 434.2357, found: 434.2354.

(4*S*,2'*R*)-3-Benzyloxycarbonyl-4-[3'-bromo-4'-(*tert*-butyldimethylsilyloxy)buta-1',2'-dienyl)-2,2-dimethyloxazolidine (15). In an oven-dried 500 mL flask with magnetic stirring bar and nitrogen inlet, *p*-TsCl (28.1 g, 148 mmol, 8 equiv) was added in one portion to a mixture of the alcohol **14** (8.0 g, 18.5 mmol) and DMAP (226 mg, 1.85 mmol, 10 mol %) in anhydrous CH_2Cl_2 (120 mL) and anhydrous pyridine (60 mL) under argon. The mixture was stirred for 17 h at rt, after which TLC control indicated complete conversion of the starting material. The solution was poured in aq sat. $NaHCO_3$ (500 mL) and the biphasic mixture was stirred until the gas evolution was finished (~60 min). The organic phase was separated and the residue was extracted with diethyl ether (3 × 100 mL). The combined organic layers were washed with brine, dried ($MgSO_4$), filtered, and the solvent was evaporated. The residue was purified by column chromatography (silica gel, isohexane/EtOAc 85:15 → 4:1) to give the tosylate (9.26 g, 85% yield) as a colorless oil. $[\alpha]^{21}_{D}$ +19.2 (*c* 1.00, $CHCl_3$); R_f = 0.73 (isohexane/EtOAc 7:3); ^{1}H NMR (400 MHz, $CDCl_3$): δ = 7.82, 7.58 (2 d, 2H, 7.40–7.20 (m, 7H, 5.65, 5.36 (2 d, 1H, 5.14 (d), 5.09 (s) (2H, 4.23–3.97 (m, 5H, 2.41, 2.38 (2 s, 3H, 1.59, 1.53, 1.48, 1.40 (4 s, 6H, 0.85 (d, 9H, 0.02 (d, 6H; ^{13}C NMR (100 MHz, $CDCl_3$): δ = 153.0, 151.9 (2 NCO_2Bn), 144.8 [*C*q–CH_3, arom. (SO_2–C_6H_4–CH_3)], 135.8, 135.7 (2 Cq, arom.), 133.4, 133.1 (2 Cq–SO_2, arom.), 129.6, 128.5, 128.2, 128.1, 128.0, 127.9 (6 CH, arom.), 95.5, 94.9 [2 *C*(CH_3)$_2$], 88.1, 88.1, 77.3, 77.1 (4 C≡C), 70.1, 69.8 (2 CH), 67.4, 67.0 (2 CH_2), 64.3, 64.0 (2 CH_2), 59.6, 58.8 (2 CH), 51.2 (CH_2), 26.4, 25.6 [2 C(*C*H_3)$_2$], 25.6 [SiC(*C*H_3)$_3$], 24.7, 23.1 [2 C(*C*H_3)$_2$], 21.5 (SO_2–C_6H_4–*C*H_3), 18.0 [Si*C*(CH_3)$_3$], –5.5 [Si(CH_3)$_2$]; IR (film): 3091, 3066, 3034, 2954, 2930, 2885, 2858, 1713, 1598, 1497, 1463, 1405, 1374, 1351, 1304, 1258, 1211, 1191, 1178, 1095, 1029, 936, 835, 814, 780, 748, 664 cm^{-1}; HRMS (ESI): *m/z* [*M* + H]$^+$ calcd for $C_{30}H_{42}NO_7S^{28}Si$: 588.2446, found: 588.2444.

Anhydrous LiBr (3.32 g, 38.3 mmol, 2.5 equiv) was transferred into a two-necked 500 mL flask with magnetic stirring bar and nitrogen inlet. The flask was evacuated and heated with a heating gun to ~300 °C for several minutes. After cooling, the flask was flushed slowly with argon and $CuBr{\cdot}SMe_2$ (7.87 g, 38.3 mmol, 2.5 equiv) and anhydrous THF (140 mL) were added. The suspension was stirred for 30 min at rt and a solution of the tosylate (9.0 g, 15.3 mmol, 1.0 equiv) in anhydrous THF (15 mL) was added by syringe. A condenser was put on the flask and the suspension was heated to 65 °C (bath temperature) giving a clear solution. After 4 h, TLC control indicated complete conversion of the starting material. The mixture was cooled to rt and the supernatant solution was decanted and concentrated under reduced pressure. Each of the residues was stirred with isohexane/diethyl ether 1:1 for 15 min and both suspensions were filtered through a pad of silica gel with suction. The filtrate was concentrated under reduced pressure and the residue was purified by column chromatography (silica gel, isohexane/EtOAc 100 → 85:15) to give the title compound **15** (4.99 g, 66%) as a mixture of diastereoisomers (*dr* = 2:1, NMR analysis). Pale yellow oil; R_f = 0.66 (isohexane/ EtOAc 4:1); ^{1}H NMR (400 MHz, $CDCl_3$): δ = 7.36 (m, 5H, 5.52 (br d, 1H, 5.27–5.07 (m, 2H, 4.58 (br m, 1H, 4.30–4.05 (m, 3H, 3.93 (dd, 1H, 1.65–1.47 (m, 6H, 0.90, 0.89 (2 s, 9H, 0.08, 0.06 (2 s, 6H; ^{13}C NMR (100 MHz, $CDCl_3$): δ = 199.5, 198.4, 198.3 (3 C=*C*=C), 152.4, 151.9, 151.8 (3 NCO_2Bn), 136.5, 136.3, 136.1 (3 Cq, arom.), 128.4, 128.0, 127.9, 127.8, 127.8 (5 CH, arom.), 100.3, 100.1, 99.5, 99.3 (4 H*C*=C=Cq), 95.0, 94.9, 94.7, 94.5, 94.1 (5 Cq), 68.1, 67.8, 67.2, 67.2, 66.8, 66.6, 65.5, 65.3 (8 CH_2), 55.8, 55.5, 55.2 (3 CHN), 27.0, 26.3, 26.1 [3 $C(CH_3)_2$], 25.7 [$SiC(CH_3)_3$], 24.8, 23.4 [2 $C(CH_3)_2$], 18.3, 18.2 [2 $SiC(CH_3)_3$], –5.3 (2 C), –5.4 [3 $Si(CH_3)_2$]; IR (film): 3091, 3066, 3033, 2983, 2953, 2930, 2884, 2875, 2710, 1976 (C=C=C), 1709, 1498, 1471, 1463, 1405, 1380, 1349,

1256, 1209, 1096, 1054, 838, 778, 766, 748, 697 cm^{-1}; HRMS (ESI): *m/z* $[M + H]^+$ calcd for $C_{23}H_{35}NO_4{}^{79}Br^{28}Si$: 496.1513, found: 496.1510.

(4*S*,2'*R*)-3-Benzyloxycarbonyl-4-[3'-bromo-4'-hydroxybuta-1',2'-dienyl)-2,2-dimethyloxazolidine (16). In a 250 mL round bottom flask, a solution of the allene **15** (4.85 g, 9.75 mmol) in THF (98 mL) was cooled to –40 °C. With stirring, *n*-$Bu_4NF·3 H_2O$ (4.46 g, 12.7 mmol, 1.3 equiv) was added in one portion and the mixture was warmed to –5 °C over 2 h, after which TLC control showed complete conversion of the starting material. The reaction was quenched by addition of aq sat. NH_4Cl (100 mL) and was diluted with water (~10 mL). The organic phase was separated and the residue was extracted with diethyl ether (3 × 80 mL). The combined organic layers were washed with brine, dried ($MgSO_4$), filtered and the solvent was evaporated. The residue was purified by column chromatography (silica gel, isohexane/EtOAc 3:2) to give the title compound **16** (3.52 g, 94%) as a mixture of diastereoisomers. Colorless oil; R_f = 0.67 (isohexane/EtOAc 1:1); ^1H NMR (400 MHz, $CDCl_3$): δ = 7.34 (m, 5H, 5.56 / 5.49* (m, 1H, 5.27–5.15 (m, 1H, 5.11–5.06 (m, 1H, 4.63 / 4.50* (m, 1H, 4.32–3.91 (m, 5H, 1.66–1.46 (m, 6H; ^{13}C NMR (100 MHz, $CDCl_3$): δ = 199.1, 197.9 (2 C=*C*=C), 153.0, 151.9 (2 NCO_2Bn), 136.1, 135.5 (2 Cq, arom.), 128.6, 128.5, 128.4, 128.2, 128.0, 127.9, 127.8, 127.7 (8 CH, arom.), 102.1, 101.0, 100.6, 99.9 (4 H*C*=C=Cq), 95.1, 94.7, 94.6, 94.0, 92.5 [5 HC=C=*C*q, $C(CH_3)_2$], 67.9, 67.6, 67.5, 67.4, 67.1, 66.9 (6 CH_2), 64.7, 64.1, 63.9 (3 CH_2), 56.0, 55.4 (2 CHN), 27.1, 26.1, 24.5, 23.3 [4 $C(CH_3)_2$]; IR (Film): 3425 (br), 3090, 3064, 3033, 2984, 2939, 2877, 1971 (C=C=C), 1703, 1498, 1455, 1409, 1383, 1351, 1254, 1209, 1145, 1095, 1054, 1030, 842, 764, 749, 698 cm^{-1}; HRMS (ESI): *m/z* $[M + H]^+$ calcd for $C_{17}H_{21}NO_4{}^{79}Br$: 382.0649, found: 382.0638.

(4*S*,2'*R*)-3-Benzyloxycarbonyl-4-(4'-amino-3'-bromobuta-1',2'-dienyl)-2,2-dimethyloxazolidine (17). Similar to the synthesis of **8a**, α-hydroxyallene **16** (3.25 g, 8.50 mmol), phthalimide (2.50 g, 17.0 mmol) and PPh_3 (4.46 g, 17.0 mmol) were stirred with DEAD (40% in toluene, 7.80 mL, 2.96 g, 17.0 mmol) in anhydrous THF (60 mL) for 30 min at 0 °C. The mixture was poured in isohexane (250 mL) to give a yellowish precipitate. Work-up as described above gave the phthalimide intermediate (3.30 g), which was refluxed with $N_2H_4{\cdot}H_2O$ (0.83 mL, 17.0 mmol) in EtOH (50 mL). After dilution with EtOAc and removal of the solvent, column chromatography (CH_2Cl_2/MeOH 20:1) gave the title compound **17** (1.44 g, 45% yield) as a mixture of diastereoisomers (*dr* = 5:1, by NMR analysis). Yellow oil; R_f = 0.70 (CH_2Cl_2/MeOH 10:1); ^{1}H NMR (400 MHz, $CDCl_3$): δ = 7.33 (m, 5H, 5.48 (m, 1H, 5.21–5.05 (m, 2H, 4.53 (m, 1H, 4.08 (dd, 1H, 3.90 (m, 1H, 3.52–3.38 (m, 1H, 3.30 (dd, 1H, 1.65–1.46 (m, 8H; ^{13}C NMR (100 MHz, $CDCl_3$): δ = 198.5, 197.8, 197.4 (3 C=*C*=C), 152.4, 151.9 (2 NCO_2Bn), 136.2, 136.0 (Cq, arom.), 128.6, 128.5, 128.1 (2 C), 127.9, 127.8 (6 CH, arom.), 101.6, 101.0, 100.5, 100.2, 100.1 (5 H*C*=C=Cq), 98.0, 96.4 (2 HC=C=*C*q), 94.7, 94.0 [2 *C*(CH_3)$_2$], 68.1, 67.6, 67.3, 66.9 (4 CH_2), 57.0, 55.9, 55.6, 55.2 (4 CHN), 48.1, 47.7, 47.5 (3 CH_2), 27.4, 27.2, 26.3, 26.2, 24.6, 24.5, 23.3 [7 C(*C*H_3)$_2$]; IR (film): 3377, 3313 (br), 3090, 3064, 3032, 2984, 2937, 2877, 1970 (C=C=C), 1703, 1498, 1455, 1405, 1381, 1350, 1252, 1208, 1094, 1054, 841, 764, 750, 698 cm^{-1}; HRMS (ESI): *m/z* [*M* + H]$^+$ calcd for $C_{17}H_{22}N_2O_3{}^{79}Br$: 381.0808; found: 381.0818.

(4*S*,2'*R*)-3-Benzyloxycarbonyl-4-(4'-bromo-2',5'-dihydro-1*H*-pyrrol-2-yl)-2,2-dimethyloxazolidine (18). In an oven-dried Schlenk tube equipped with a magnetic stirring bar, α-aminoallene **17** (144 mg, 0.38 mmol), imidazole (2.6 mg, 0.04 mmol, 10 mol %) and AuCl (8.8 mg, 0.04 mmol, 10 mol %) were dissolved in anhydrous

toluene (1.5 mL) under argon. The tube was sealed with a stopper and the yellow solution was stirred for 16 h at 100 °C. After cooling, the mixture was concentrated under reduced pressure and the residue was purified by column chromatography (silica gel, CH_2Cl_2/MeOH 20:1) to give the title compound **18** (97 mg, 67%) as a brown liquid and contaminated with impurities (~5%, by NMR analysis). $[\alpha]^{19}_{D}$ +65.2 (*c* 0.86, $CHCl_3$); R_f = 0.83 (CH_2Cl_2/MeOH 10:1); ^1H NMR (400 MHz, $CDCl_3$): δ = 7.36 (m, 5H, 5.89 (d, 1H, 5.14 (m, 2H, 4.43 (2 s, 1H, 4.02–3.77 (m, 4H, 2.17 (br s, 1H, 1.66, 1.58, 1.50, 1.43 (4 s, 6H; ^{13}C NMR (100 MHz, $CDCl_3$): δ = 153.8, 152.7 (2 NCO_2Bn), 136.3, 136.0 (2 Cq, arom.), 128.6, 128.5, 128.3, 128.2, 128.0 (5 CH, arom.; H*C*=C_q), 119.7, 119.3 (HC=*C*q), 94.9, 94.3 [2 *C*(CH_3)$_2$], 67.4 (CH_2), 67.1 (CH), 66.8 (CH_2), 66.8 (CH), 64.3, 64.0 (2 CH_2), 61.8, 60.8 (2 CHN), 58.8 (CH_2), 27.2, 26.2, 24.1, 22.6 [4 C(CH$_3$)$_2$]; IR (film): 3365, 3089, 3063, 3032, 2984, 2939, 2878, 1699, 1624, 1497, 1455, 1437, 1406, 1380, 1349, 1256, 1207, 1149, 1095, 1055, 1029, 837, 764, 750, 733, 697, 544 cm^{-1}; HRMS (ESI): *m/z* [*M* + H]$^+$ calcd for $C_{17}H_{22}N_2O_3{}^{79}Br$: 381.0808; found: 381.0819.

(4*S*,2’*R*)-3-Benzyloxycarbonyl-4-(1’-benzyloxycarbonyl-4’-bromo-2’,5’-dihydro-1*H*-pyrrol-2-yl)-2,2-dimethyloxazolidine (19). Similar to the preparation of compounds **11a** and **11b**, in a 100 mL round-bottom flask with magnetic stirring bar, CbzCl (768 µL, 921 mg, 5.40 mmol, 3 equiv) was added to a vigorously stirred solution of dihydropyrrole **18** (690 mg, 1.80 mmol) and DMAP (660 mg, 5.40 mmol, 3 equiv) in acetonitrile (30 mL) at rt. After 45 min with stirring, the solution was poured in aq sat. $NaHCO_3$ (50 mL) and the mixture was extracted with diethyl ether (4 × 30 mL). The combined organic layers were washed with brine, dried ($MgSO_4$), filtered and the solvent was evaporated. The residue was purified by column chromatography (silica gel, isohexane/EtOAc 85.15 → 4:1) to give the title compound

19 (540 mg, 58%; 42% over two steps starting from allene **17**) as a colorless oil. $[\alpha]^{19}_{D}$ +37.9 (*c* 1.27, $CHCl_3$); R_f = 0.60 (isohexane/EtOAc 7:3); HRMS (ESI): *m/z* [*M* + H]$^+$ calcd for $C_{25}H_{28}N_2O_5{}^{79}Br$: 515.1176; found: 515.1172. The spectroscopic data were in full agreement with those of the enantiomer (4*R*,2'*S*)-**11b**.

(4*S*,2'*R*)-3-Benzyloxycarbonyl-4-(1'-benzyloxycarbonyl-2',5'-dihydro-1*H*-pyrrol-2-yl]-2,2-dimethyloxazolidine (20). In an oven-dried Schlenk tube equipped with a magnetic stirring bar, dihydropyrrole **19** (225 mg, 0.44 mmol) was dissolved in anhydrous diethyl ether (4.4 mL) under argon. After cooling to –90 °C, *t*-BuLi (1.6 M in pentane, 550 µL, 0.88 mmol, 2 equiv) was added dropwise. After 5 min with stirring, water (1.0 mL) was added and the cooling bath was removed. After dilution with water (10 mL) and diethyl ether (5 mL), the organic phase was separated and the residue was extracted with diethyl ether (3 × 10 mL). The combined organic layers were washed with brine, dried ($MgSO_4$), filtered and the solvent was evaporated. The residue was purified by column chromatography (silica gel, isohexane/EtOAc 4:1 → 7:3) to give reisolated compound **19** (49 mg) and the title compound **20** (115 mg, 60%) as a pale yellow oil. $[\alpha]^{20}_{D}$ +13.6 (*c* 1.00, $CHCl_3$); R_f = 0.38 (isohexane/EtOAc 7:3); ^1H NMR (400 MHz, $CDCl_3$): δ = 7.53–7.25 (m, 10H, 5.93 (dd, 1H, 5.75 (m, 1H, 5.27–5.01 (m, 4H, 4.89–4.45 (m, 2H, 4.31 (m, 1H, 4.14 (m, 1H, 3.86 (m, 1H, 3.64 (m, 1H, 1.65–1.57 (m, 3H, 1.47–1.41 (m, 3H; ^{13}C NMR (100 MHz, $CDCl_3$): δ = 154.8, 154.6, 152.8, 152.7 (4 NCO_2Bn), 136.5, 136.4, 136.4 (3 Cq, arom.), 128.4, 128.0, 127.9, 127.8, 127.7, 127.7, 127.6 (7 CH, arom.), 126.9, 126.8, 126.6, 126.4 (4 H*C*=CH), 94.9 [*C*(CH_3)$_2$], 66.9, 66.8, 66.6, 66.6 (4 CH_2), 65.2, 64.8, 64.5, 63.8 (4 CH), 63.5, 63.4 (2 CH_2), 58.9, 58.2, 57.9, 57.0 (4 CH), 54.9, 54.4 (2 CH_2), 26.6, 26.5, 25.6, 24.1, 24.0, 22.5 [6 C(*C*H_3)$_2$]; IR (film): 3090, 3065, 3032, 2985, 2941, 2874, 1704, 1498, 1455, 1409, 1361, 1332, 1280, 1259, 1226, 1207,

1151, 1103, 1053, 1009, 841, 769, 751, 698, 602 cm^{-1}; HRMS (ESI): *m/z* [*M* + H]$^+$ calcd for $C_{25}H_{29}N_2O_5$: 437.2071; found: 437.2070.

(2*R*,1’*S*)-*N*-Benzyloxycarbonyl-2-(1’-benzyloxycarbonyl-2’,5’-dihydropyrrol-2-yl)-glycine (21). In a round-bottom flask, a solution of **20** (175 mg, 0.40 mmol) and *p*-TsOH·H_2O (15 mg, 0.08 mmol, 0.2 equiv) in MeOH (4 mL) was stirred overnight at rt. Further *p*-TsOH·H_2O (45 mg, 0.24 mmol, 0.6 equiv) was added and stirring was continued for 20 h. A third portion *p*-TsOH·H_2O was added (15 mg, 0.08 mmol, 0.2 equiv) and stirring was continued for 4 h. After 40 h total time of stirring, the solvent was removed in vacuo, and the residue was filtered by a short column (silica gel, isohexane/EtOAc 1:1) to give the hydroxycarbamate (113 mg, 71%) as a colorless oil. $[\alpha]^{20}_{D}$ +117.4 (*c* 2.60, $CHCl_3$); R_f = 0.23 (isohexane/EtOAc 1:1); ^{1}H NMR (400 MHz, $CDCl_3$): δ = 7.34 (m, 10H, 5.75 (m, 2H, 5.16–5.05 (m, 5H, 4.96 (s, 1H, 4.35–4.22 (m, 2H, 4.03–3.94 (m, 2H, 3.73 (m, 1H, 3.51 (m, 1H; ^{13}C NMR (100 MHz, $CDCl_3$): δ = 157.1, 156.3 (2 NCO_2Bn), 136.3, 135.9 (2 Cq, arom.), 128.5, 128.3, 128.2, 128.0, 127.9, 127.9 (6 CH, arom.; HC=CH), 126.1 (HC=CH), 67.6, 66.6 (2 CH_2), 65.7 (CH), 62.5 (CH_2), 55.3 (CHN), 54.7 (CH_2); IR (film): 3411 (br), 3323 (br), 3089, 3064, 3033, 2951, 2873, 1701, 1626, 1534, 1498, 1454, 1417, 1384, 1363, 1318, 1291, 1242, 1215, 1105, 1056, 1028, 973, 772, 738, 697 cm^{-1}; HRMS (ESI): *m/z* [*M* + H]$^+$ calcd for $C_{22}H_{25}N_2O_5$: 397.1758; found: 397.1750.

Similar to the preparation of **12a**, the hydroxycarbamate (108 mg, 0.27 mmol) was stirred with Dess–Martin periodinane [7,8] (390 mg, 0.92 mmol, 3.4 equiv) in CH_2Cl_2 (3.0 mL) for 2 h at 0 °C. Work-up as described above gave the crude aldehyde (108 mg), which was treated with $NaClO_2$ (121 mg, 1.07 mmol) and NaH_2PO_4·H_2O (148 mg, 1.07 mmol) in dioxane (25 mL) in the presence of resorcine (40 mg, 0.36 mmol). Work-up as described above and column chromatography gave the title compound

21 (80 mg, 72%) as a colorless oil. $[\alpha]^{20}_{D}$ +87.8 (*c* 1.76, $CHCl_3$); R_f = 0.30 (isohexane/EtOAc/AcOH 7:3:1); ^1H NMR (400 MHz, $CDCl_3$): δ = 9.71 (br s, 1H, 7.33 (m, 10H, 6.17–5.59 (m, 3H, 5.34–4.97 (m, 5H, 4.71, 4.56 (2 d, 1H, 4.28–4.02 (m, 2H; ^{13}C NMR (100 MHz, $CDCl_3$): δ = 173.9, 173.7 (2 COOH), 156.3, 156.0, 155.6, 154.8 (4 NCO_2Bn), 136.2, 136.1, 135.9 (3 Cq, arom.), 128.4, 128.3, 128.1, 128.0, 127.9, 127.8 (6 CH, arom.), 127.3, 127.1 (2 HC=CH), 67.5, 67.3, 67.2 (3 CH_2), 67.0 (CH), 66.9 (CH_2), 65.9 (CH), 56.6, 56.0 (2 CH), 54.8, 54.3 (2 CH_2); IR (film): 3411 (br), 3311 (br), 3090, 3065, 3033, 2953, 2872, 2586 (br), 1709, 1625, 1515, 1500, 1455, 1418, 1384, 1364, 1327, 1237, 1215, 1123, 1111, 1060, 753, 697 cm^{-1}; HRMS (ESI): *m/z* $[M + H]^+$ calcd for $C_{22}H_{23}N_2O_6$: 411.1551; found: 411.1547.

(α*S*,2*R*)-(2’,5’-Dihydro-1*H*-pyrrol-2-yl)glycine (22). In an apparatus consisting of a25 mL round-bottom flask and a condenser, TFA (7.1 mL, 91.8 mmol) was added to a mixture of **21** (70 mg, 0.17 mmol) and thioanisole (2 mL, 17.0 mmol). It was stirred overnight at rt (~13 h) followed by heating to 50 °C (bath temperature) for 2 h. Volatile components were removed in oil pump vacuum and the dark residue was passed through a column of ion exchange resin (DOWEX 50W X8, length 10 cm, diameter 1.7 cm) to give 30 mg of crude product. To get an analytically pure product, ion exchange chromatography was repeated twice to give the title compound **22** (16 mg, 66%) as an amorphous, pale yellow solid. Mp 180–185 °C (dec), $[\alpha]^{22}_{D}$ +57 (*c* 0.20, H_2O); ^1H NMR (400 MHz, D_2O): δ = 6.05 (dd, *J* = 4.5 Hz, 1H 3-H), 5.81 (dd, *J* = 4.4 Hz, 1H 4-H), 4.68 (br s, 1H 2-H), 3.96 (s, 2H 5-H), 3.72 (d, *J* = 4.0 Hz, 1H α-H); ^{13}C NMR (100 MHz, D_2O): δ = 175.6 (COOH), 129.4 (HC=CH), 125.6 (HC=CH), 66.9 (CHN), 56.2 (CH), 52.8 (CH_2); IR (KBr): 3439 (br), 3356, 3073, 2952, 2891, 2864, 2839, 2491 ($–NH_3^+$), 2285 (br), 1622, 1396, 1356, 1337, 520 cm^{-1}; HRMS (ESI): *m*/*z* $[M + H]^+$ calcd for $C_6H_{11}O_2N_2$: 143.0815; found: 143.0808.

References

1. Love, B. E.; Jones, E. G. *J. Org. Chem.* **1999**, *64*, 3755.
2. Kofron, W. G.; Baclawski, L. M. *J. Org. Chem.* **1976**, *41*, 1879.
3. Tsou, H.-R.; Mamuya, N.; Johnson, B. D.; Reich, M. F.; Gruber, B. C.; Ye, F.; Nilakantan, R.; Shen, R.; Discafani, C.; DeBlanc, R.; Davis, R.; Koehn, F. E.; Greenberger, L. M.; Wang, Y.-F.; Wissner, A. *J. Med. Chem.* **2001**, *44*, 2719.
4. Hassall, C. H.; Thomas, J. O. *J. Chem. Soc.* (C) **1968**, 1495.
5. Chhabra, S. R.; Mahajan, A.; Chan, W. C. *J. Org. Chem.* **2002**, *67*, 4017.
6. McKillop, A.; Taylor, R. J. K.; Watson, R. J.; Lewis, N. *Synthesis* **1994**, 31.
7. Dess, D. B.; Martin, J. C. *J. Org. Chem.* **1983**, *48*, 4155.
8. Boeckmann Jr., R. K.; Shao, P.; Mullins, J. *Org. Synth.* **2004**, *Coll. Vol. 10*, 696.
